# A comprehensive analysis of nanomagnetism models for the evaluation of particle energy in magnetic hyperthermia

**DOI:** 10.1039/d5na00258c

**Published:** 2025-05-27

**Authors:** N. Maniotis, M. Maragakis, N. Vordos

**Affiliations:** a Department of Physics, Aristotle University of Thessaloniki Thessaloniki Greece nimaniot@physics.auth.gr; b Department of Physics, Democritus University of Thrace Kavala Greece

## Abstract

Magnetic nanoparticles (MNPs) have attracted significant research interest due to their unique magnetic properties, which differ from their bulk counterparts and enable applications in information technology, environmental protection, and biomedicine. Among these applications, magnetic particle hyperthermia (MPH) has emerged as a promising therapeutic approach for cancer treatment. This review provides a comprehensive analysis of nanomagnetism models used to evaluate the heating potential of MNPs in MPH. Specifically, we examine (i) theoretical approaches for estimating the magnetic properties of nanoparticle systems and (ii) numerical simulation strategies that predict their response to externally applied magnetic fields. Common modeling frameworks typically focus on key magnetic parameters such as total energy, magnetization, anisotropy, and hysteresis loop morphology. However, precise characterization of these properties remains challenging due to their dependence on multiple interrelated factors, including particle size, shape, composition, and interparticle interactions. To address these challenges, this review discusses various analytical and numerical models that aid in the qualitative and quantitative assessment of MNP behavior under alternating magnetic fields. By critically evaluating these methodologies, we aim to enhance the understanding of magnetic field-driven heating mechanisms and contribute to the optimization of MNPs for hyperthermia-based therapeutic applications. Looking forward, the integration of advanced multiphysics simulations, combining magnetization dynamics with biological, thermal, and fluidic environments, is anticipated to revolutionize the predictive accuracy and translational potential of MPH technologies.

## Abstract 2

Magnetic nanoparticles have attracted significant research interest due to their unique magnetic properties, which differ from their bulk counterparts and enable applications in information technology, environmental protection, and biomedicine.

## Graphical abstract

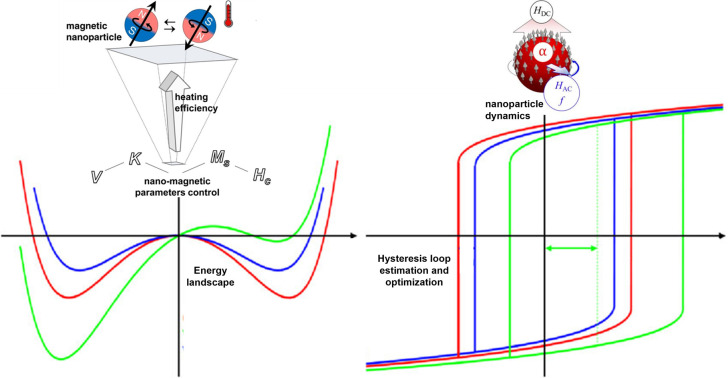

## Introduction

Magnetic particle hyperthermia (MPH) is an innovative and minimally invasive cancer treatment that utilizes a magnetic fluid, also known as ferrofluid, as a localized heat source.^1–4^ This ferrofluid consists of a stable colloidal suspension of magnetic nanoparticles (MNPs), which can be directly injected into the tumor or delivered through intravenous administration *via* passive or active (functionalized) targeting.^5,6^ Those systems are usually of iron oxide nanoparticles that are suspended – very finely distributed – in water. After administration, magnetic nanoparticles can be directed toward the tumor site either through passive targeting mechanisms—such as the enhanced permeability and retention (EPR) effect—or *via* active targeting strategies involving surface functionalization with ligands that bind to tumor-specific receptors.^7^ Once accumulated to the tumor area, nanoparticles are exposed to an external alternating magnetic field (AMF) that causes reversal of their magnetic moments, activating mechanisms of energy transfer in the form of heat.^8,9^

Due to their unique properties, MNPs have gained significant research attention which emerges from their nanoscale dimensions and differentiates them from their bulk counterparts. Beyond the advantages of miniaturization in high-tech applications and enhanced surface-area-to-volume ratios in conventional technologies,^10–13^ MNPs exhibit a novel magnetic behavior driven by their reduced dimensionality.^14–16^ As the size of a material approaches the nanometer scale, surface effects become increasingly substantial, often rivaling or surpassing bulk contributions.^17–19^ In order to ensure both stability and biocompatibility, MNPs are commonly coated or functionalized with biocompatible materials such as polyethylene glycol (PEG), dextran, or starch.^16,20,21^ These coatings not only stabilize the particles by reducing surface energy and preventing agglomeration but also improve their dispersion and circulation time in biological environments.^22^ Furthermore, structural defects resulting from broken crystalline symmetry play a crucial role in determining magnetic properties, while additional physical effects emerge when the particle size reaches the material's intrinsic characteristic length scales. These size-dependent effects make MNPs highly sensitive to variations in size, shape, and chemical composition, necessitating specialized theoretical and computational approaches to accurately describe their behavior.^23–26^

The collective magnetic properties of MNPs arise from a complex interplay between intrinsic parameters such as the magnetic moment and anisotropy and external experimental conditions like the applied magnetic field and the measurement timescale.^27^ While significant progress has been made in understanding these properties, accurately modeling the dynamic response of MNPs under alternating magnetic fields—particularly in the context of MPH—remains a challenge. The difficulty primarily stems from the wide range of relevant length and time scales involved, making conventional atomistic simulations computationally demanding and often impractical.^28^ Moreover, large-scale simulations of dense nanoparticle systems encounter limitations due to the complex interactions between particles and externally applied magnetic fields, as well as the lack of universally reliable models, valid for these type of interactions.^29–32^

A key challenge in simulating realistic MNP systems is that neither atomic-scale models nor continuum-based approaches alone can fully capture the intricate magnetic behaviors at intermediate scales. The mesoscopic regime—spanning approximately from 10^−9^ to 10^−6^ meters—bridges the gap between atomistic simulations and macroscopic continuum models.^33,34^ Within this scale, individual nanoparticle dynamics are still relevant, yet the system is too large to be efficiently simulated using purely atomistic methods. Mesoscopic modeling provides an effective way to address this challenge by considering local equilibrium at the microscopic level while capturing emergent behaviors on longer timescales.^35^ One of the most widely used techniques at this scale is micromagnetic modeling,^36^ which provides a phenomenological framework for determining equilibrium magnetization configurations in MNPs based on applied field conditions,^37^ particle geometry,^38^ and material properties.^39^ The field of numerical micromagnetics continues to evolve, with ongoing advancements improving its predictive capabilities.^40,41^

Given the complexity of MNP interactions and their strong dependence on microstructural and morphological factors, a combination of analytical and numerical approaches is necessary to effectively model their heating potential in hyperthermia applications. This review provides a comprehensive analysis of mesoscopic models designed to estimate and optimize key magnetic properties relevant to MPH, including anisotropy, total energy, magnetization, and hysteresis loop area. Special emphasis is placed on numerical simulations, which enable the study of non-local magnetic interactions and dynamic processes that cannot be resolved analytically. By critically assessing these modeling strategies, we aim to enhance the understanding of field-driven heating mechanisms in MNPs, ultimately contributing to the development of more efficient hyperthermia-based therapies.

## Single particle approximations: analytical models in MPH

When magnetic nanoparticles are exposed to an external AMF, as used in MPH, the total energy they acquire primarily originates from two key contributions. Firstly, there is the Zeeman energy, which corresponds to the energy transferred to the nanoparticles by the external field. Secondly, there is the anisotropy energy, which arises due to the deviation of the nanoparticle's magnetic moment from its preferred easy-axis direction, under the influence of the alternating field. This misalignment imposes an additional energy cost on the system. Since MNPs in MPH applications experience continuously changing magnetic fields, their magnetization dynamics is translated to hysteresis losses, which contribute to the dissipation of energy as heat, an essential feature for hyperthermia treatment. Analyzing the total energy involved in this process requires first considering a single nanoparticle, before extending the analysis to an ensemble.

**Fig. 1:**
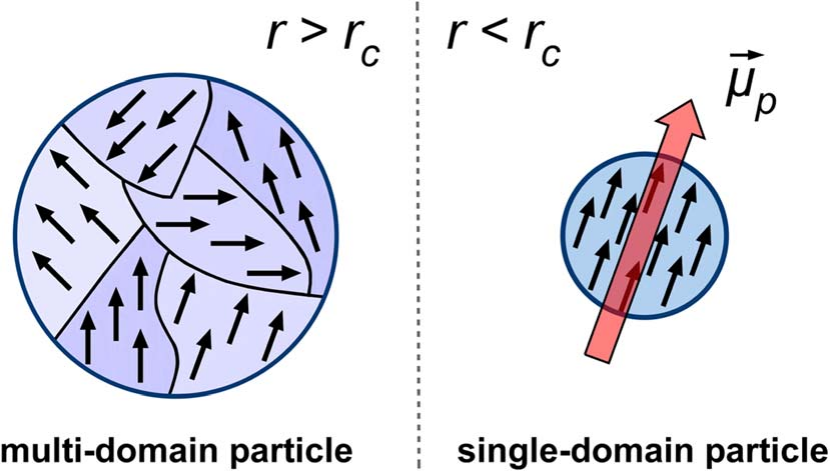
Schematic representation of magnetic domain structures in nanoparticles based on their size. Particles with a radius *r* larger than a critical value *r*_c_ exhibit a multi-domain structure (left), where multiple magnetic domains are separated by domain walls. When the particle radius is smaller than *r*_c_, it becomes a single-domain particle (right), where all atomic spins align coherently, resulting in a net magnetic moment *μ⃗*_p_ in a single direction.^43^

Let us consider a ferromagnetic nanoparticle of volume *V*, with a saturation magnetization *M*_s_ and an effective anisotropy constant *K*_eff_. Unlike bulk ferromagnetic materials that exhibit multi-domain structures, nanoparticles below a critical radius *r*_c_ (which depends on the material and its shape) exist in a single-domain state to minimize their total energy (Fig. 1). Since the critical dimension for most materials falls within the nanometer range, MNPs used in MPH are typically assumed to be single-domain entities. Their energy dissipation, governed by hysteresis losses and relaxation mechanisms, plays a crucial role in determining their heating efficiency under an AMF.

In the single-domain model, all spins are parallel to one another and the magnetic moment *μ* can be described as a single giant magnetic moment:

1 |*μ*|=*M*_s_*V*

the amplitude of which is independent of its spatial orientation. Eqn (1) depicts the so-called “macrospin” and “coherent rotation” approximations.^42^

Another approximation is that the magnetic nanoparticle is assumed to have a uniaxial magnetocrystalline anisotropy according to which the crystal system has a single-axis of high symmetry. Assuming these approximations, the energy of a magnetic nanoparticle placed in an AMF *H* is given by the following equation which was first introduced by Stoner and Wohlfarth:^44^

2 *E*(*θ*,*φ*) = *K*_eff_*V* sin^2^(*θ*) − *μ*_0_*M*_s_*VH* cos(*θ* − *φ*)

with *θ* the angle between the easy axis and the magnetic moment *μ* and *φ* the angle between the “easy” axis and the magnetic field *H*. The first term in eqn (2) represents the energy contribution due to the effective anisotropy of the nanoparticle while the second term, known as Zeeman energy, is proportional to nanoparticle's energy originating by the interaction with the AMF. The latter is a harmonic field that can be written as *H* = *H*_0_ sin(2π*ft*), where *H*_0_ and *f* are the field amplitude and frequency respectively. According to the Stoner–Wohlfarth model, *E*(*θ*) behavior can be estimated by assuming zero thermal effects (*i.e.* at *T* = 0 K) and a constant “easy” axis angle with the magnetic field orientation (specific value of *φ*) which is a reasonable hypothesis for a single particle. Normally, eqn (2) is solved through energy minimization processes without applying time-dependent temperature variations. In order to take into account the thermal energy contribution *k*_B_*T* at temperatures higher than 0 K in the energy solution, the dimensionless parameters 
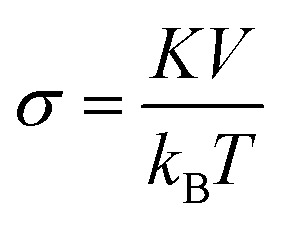
 and 
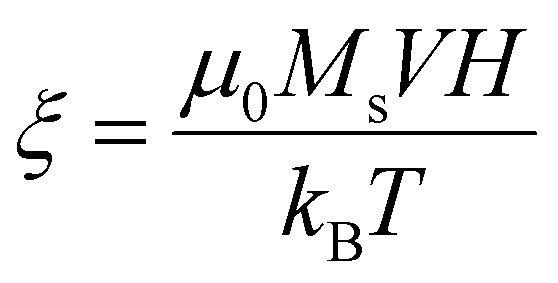
 are introduced. The parameter *σ* is usually referred as anisotropy barrier. Then, the reduced magnetic energy normalized to *k*_B_*T* is:3
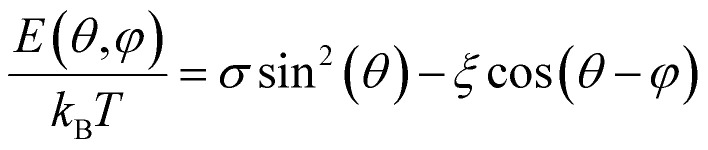


If the external magnetic field is applied along the “hard” axis (*φ* = π/2), then the energy is expressed as *E* = *K* sin^2^ *θ* + *μ*_0_*HM*_s_ sin *θ*, and the angle *θ* that minimizes the energy in this case is found by:
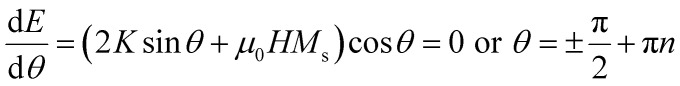
and
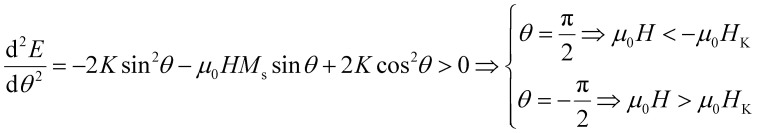


**Fig. 2:**
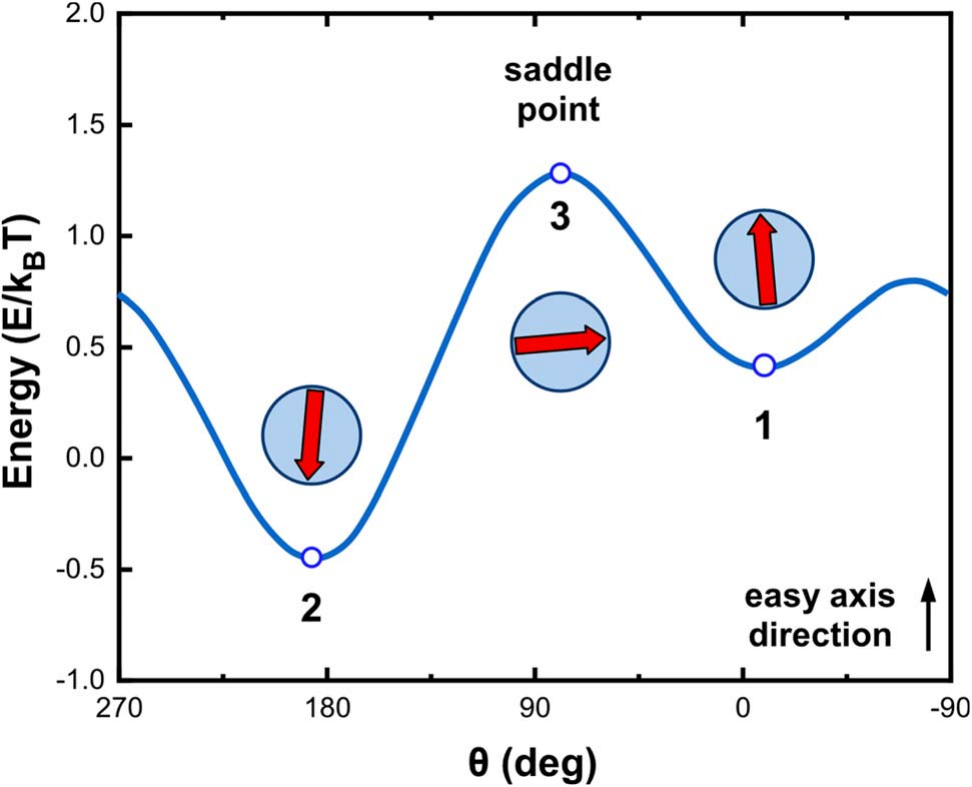
Schematic representation of the energy landscape variation with the “easy” axis-magnetic moment angle (double-well approximation) for *ξ* = 0.5 and *φ* = 30°. The red arrows show the magnetic moment orientation. When *ξ* ≠ 0, *θ*_1_, *θ*_2_, and *θ*_3_ values deviate slightly from 0, 180 and 90 degrees which correspond to parallel antiparallel and perpendicular orientation, with respect to the “easy” axis, respectively. Although this deviation exists, we can approximately employ 0 and 180 degrees as the minimum energy positions without a significant deviation from the realistic experimental behavior.^45^

The unique way that these conditions are satisfied is when 
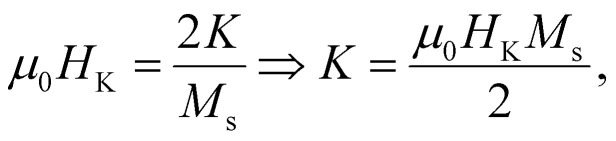
 where *μ*_0_*H*_K_ corresponds to the anisotropy field representing the field at which *μ* reaches its maximum value *i.e.* reaches saturation. When the applied magnetic field *μ*_0_*H* is higher than the anisotropy field, the energy landscape displays only one minimum, which defines the equilibrium position, *i.e.*, along the anisotropy axis direction. Conversely, when *μ*_0_*H* is lower than *μ*_0_*H*_K_, the energy profile as a function of *θ* displays two minima at positions (*θ*_1_, *E*_1_), (*θ*_2_, *E*_2_) and two maxima as depicted in Fig. 2 for *φ* = 30°. The upper maximum point at position (*θ*_3_, *E*_3_) is known as the saddle point.

As described, the total energy minimization and the estimation of *E*(*θ*) behavior can be done analytically in the case of a single nanoparticle. However, in the case of an assembly of magnetic nanoparticles, where the system is characterized by a random distribution in *φ* values, this analytical solution is not valid and computational techniques should be utilized.^43,46^ On this, the most frequently used technique is the Monte Carlo (MC) minimization method. This simulation approach is based on the generation of random numbers (random sampling) to obtain numerically the minimization of a given energy functional. In magnetic nanoparticles, the random numbers coincide with “easy” axis orientations because they are used to generate trial orientations of the magnetization vector relative to the easy axis and the minimization proceeds for the energy function *E*(*θ*,*φ*) given by eqn (2). The described methodology was employed in a characteristic system of iron oxide (Fe_3_O_4_) magnetic nanoparticles^47^ at room temperature *T* = 300 K, for magnetic fields amplitudes typically used in MPH and a frequency equal to 300 kHz. Since the applied magnetic field is alternating, the considered case is essentially quasi-static, and therefore, the conditions ruling the system are expressed by an energy minimization processes.

**Fig. 3:**
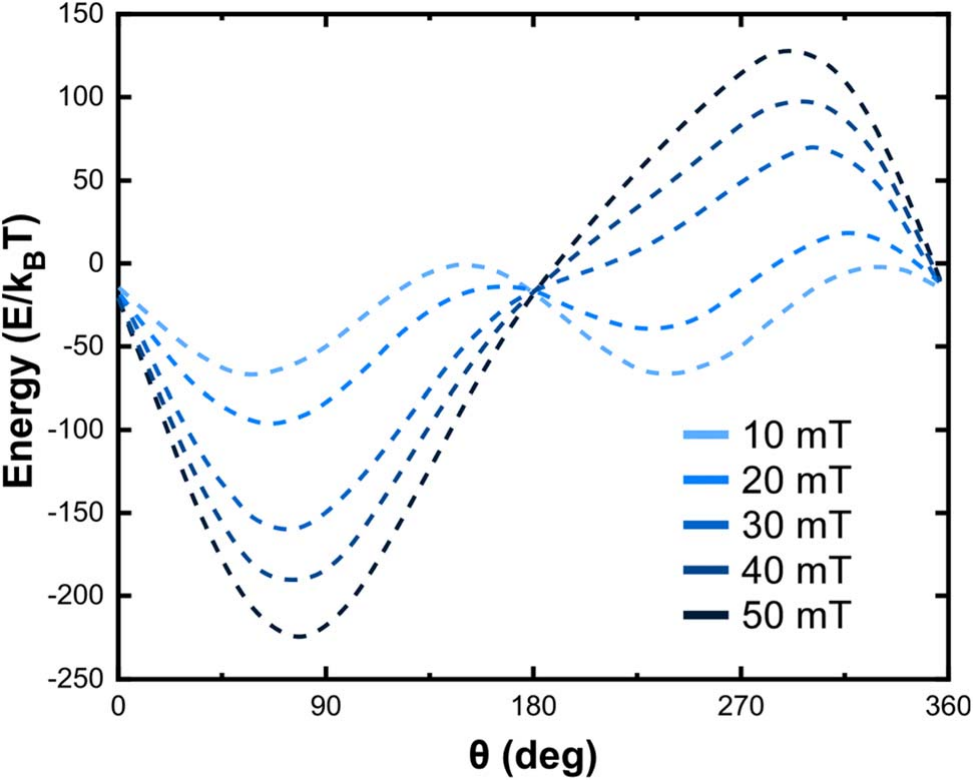
Magnetic energy as a function of angle *θ* for various values of *μ*_0_*H* at *T* = 300 K and *f* = 300 kHz. The results were obtained after energy minimization with the Monte Carlo method for a single Fe_3_O_4_ magnetic nanoparticle.^45^ The transformation of the energy profile under increasing external magnetic field strength is visually demonstrated by clearly showing the disappearance of the metastable state and the emergence of a single energy minimum aligned with the easy axis.

The results of the above procedure are more clearly comprehended by Fig. 3 where the *E*/*k*_B_*T* ratio is plotted *versus θ* for various values of *H* in the range typically used in biomedical applications and more specifically for magnetic hyperthermia protocols.^48–50^ For *ξ* = 0, a value that corresponds to the absence of magnetic fields or for infinite temperatures, *μ* can take two equivalent equilibrium values at *θ*_1_ = 0° and *θ*_2_ = 180°, *i.e.*, along its “easy” axis. For a finite positive value of *ξ*, the magnetic field favors one of the two minima. Increasing *ξ*, moves the abscissa of this minimum progressively so that the parallel orientation of *μ* to the magnetic field is favored. In the example shown in Fig. 3, when the applied magnetic field is higher than the anisotropy field, the energy landscape (as a function of the angle *θ* between easy axis and *μ*) displays only one minimum, which defines the equilibrium position, *i.e.*, along the anisotropy axis direction (curves corresponding to an applied magnetic field amplitude of 40 and 50 mT in Fig. 3). Conversely, when the applied magnetic field is smaller than the anisotropy field, the energy profile as a function of *θ* displays two minima and two maxima (curves corresponding to an applied magnetic field amplitude of 10 and 20 mT).

**Fig. 4:**
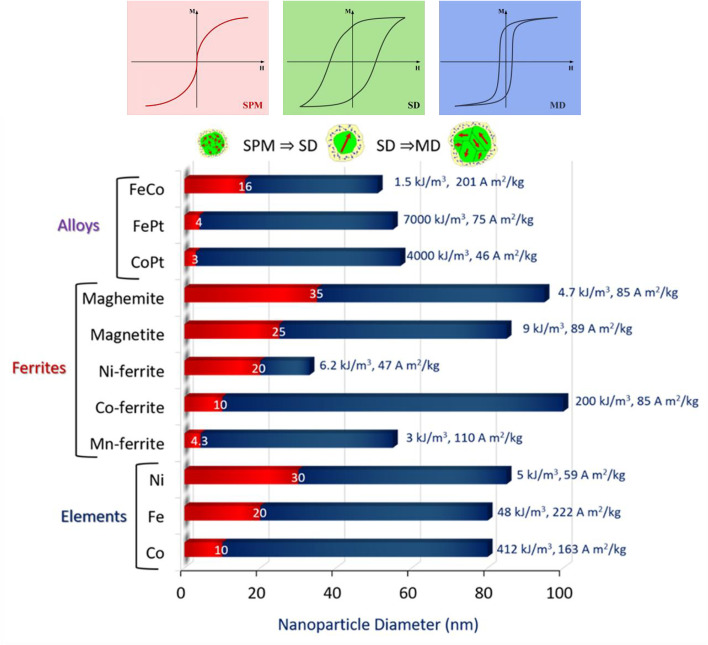
Nanomagnetic size effects: top graphs outline the collective magnetic features exhibited by the hysteresis loops and spin configurations in each one of the three regions with surface spins also sketched at the outer layer of the particle. Main graph shows the magnetic transitions occurring as MNPs grow in diameter. transition area between red and blue bars corresponds to transition from (SPM) superparamagnetism to (SD) ferromagnetism-single-domain particles while the rightmost edge of blue bars corresponds to the critical diameter above which the formation of (MD) multiple domains is energetically favored. Next to the bar charts effective anisotropy and room temperature saturation magnetization values are given as collected from ref. 47.

In order to calculate the total energy, we examined the case where nanoparticles have reached saturation and, thus, magnetization was assumed to be independent to the applied magnetic field *H* and equal to a constant value *M*_s_. In order to expand the validity of this approach and thoroughly study the magnetic properties of nanoparticles, the dependence of magnetization on *H* should be introduced through analytical relationships of the *M*(*H*) curve. First, let us consider a case of zero or very low anisotropy barrier, *i.e. K*_eff_*V*/*k*_B_*T* ≪ 1 and *K*_eff_ ≤ 1 kJ m^−3^ where the virgin magnetization curve is given by:

4 *M*(*H*) = *M*_s_*L*(*ξ*)

with 
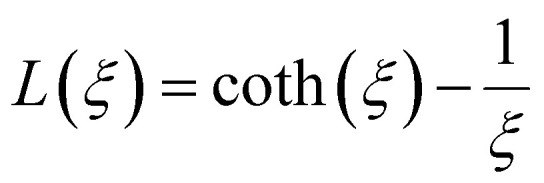
 being the Langevin function which intrinsically neglects the anisotropy of magnetic nanoparticles and introduces the Zeeman term as the main contribution of the total energy in eqn (2). Eqn (4) is valid in the case of very small nanoparticles with dimensions lying in the superparamagnetic regime which are characterized by a low *K*_eff_*V* product. On the other hand, the Langevin function fails to describe the magnetization of magnetic nanoparticles with effective anisotropy above 1 kJ m^−3^ and/or large nanoparticles with dimensions lying in the superparamagnetic to ferromagnetic transition range. Using theories for domain stability in fine particles and bulk properties available in the literature, one can determine the characteristic size up to which single-domains are stable. This series of magnetic “phases” as a function of size is shown in Fig. 4 for different ferromagnets, which includes a “single-domain” size *D*_sd_, below which the material will not support a multi-domain particle, and a size *D*_sp_ defined by the superparamagnetic effect, below which a spontaneous flip in magnetization occurs due to thermal effects at room temperature.

For MPH applications, it is a prerequisite to know the magnetic status of the MNPs since the energy release and thus the heating mechanism differs for particles possessing or not hysteresis.

The second case emerges for 
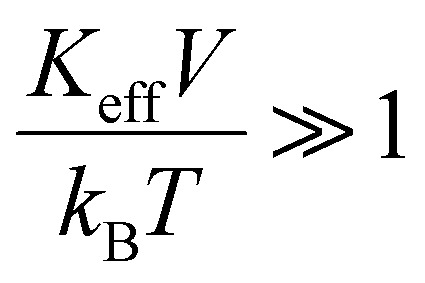
 and *μ*_0_*H*_K_ > *μ*_0_*H* and assumes the neglection of excited energy states inside each energy well, therefore, magnetization calculations only depend on the position of the two energy minima *E*_1_ and *E*_2_ and the energy maximum *E*_3_ as depicted in Fig. 2. This approach is known as the double-well (DW) approximation and has been applied not only in the study of magnetic nanosystems,^51,52^ but also in many fields of physics such as quantum mechanics,^53–55^ neural networks^56,57^ and protein structure prediction.^58–60^ The values of *E*_1_, *E*_2_ and *E*_3_ are obtained after solving eqn (2) either for random or parallel (*φ* = 0) orientation of the magnetic field with respect to the “easy” axis. When applied magnetic field is below *μ*_0_*H*_K_, the magnetization can switch from the one minimum to the other at a rate *ν*_1_ expressed by:5
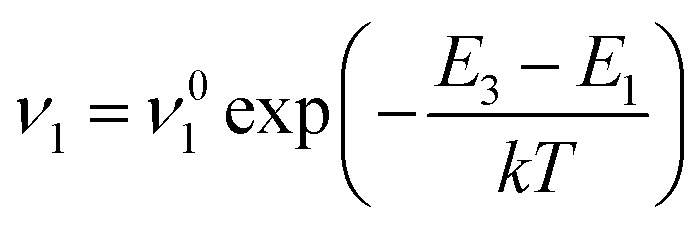


Similarly, the switching rate *ν*_2_ from the opposite direction is given by:6
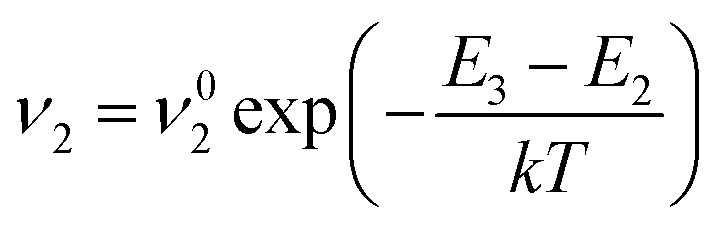
where the frequencies *v*^0^_1_ and *v*^0^_2_, under specific assumptions, can be related to the Larmor frequency which is given as a function of both material parameters (gyromagnetic ratio, damping, saturation magnetization and anisotropy constant) and experimental conditions (temperature and magnetic field strength). An approximate expression for the Larmor precession frequency *ν*_L_ to reflect this dependency is 
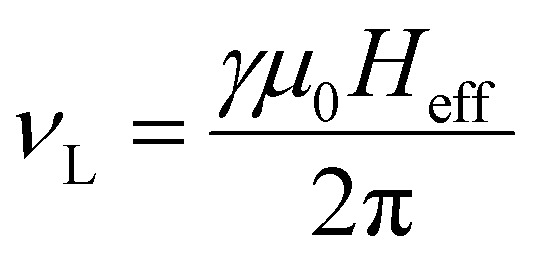
 where *γ* is the gyromagnetic ratio and *H*_eff_ is the effective magnetic field, which encompasses contributions from the anisotropy field, external field, and internal demagnetizing fields. For the sake of simplicity, these frequencies are kept constant and equal to 1/*τ*_0_, where *τ*_0_ is a typical time value for ferromagnetic resonance^61^ set equal to 10^−10^ s. In the case of small magnetic field amplitudes *H i.e.* low values of *ξ* (as in MPH application) the two energy minima can be approached as *θ*_1_ = 0° and *θ*_2_ = 180°. Thus, the energy differences Δ*E*_1_ and Δ*E*_2_ between each minimum and the saddle point can estimated by eqn (5) and (6), for the case of *φ* = 0, as Δ*E*_2,1_ = *K*_eff_*V*(1 ± *H*_0_/*H*_K_)^2^. Calling *P*_1_ the occupation probability of position *E*_1_ and *P*_2_ the corresponding one of position *E*_2_, the time evolution of *P*_1_ is estimated by:^62^7
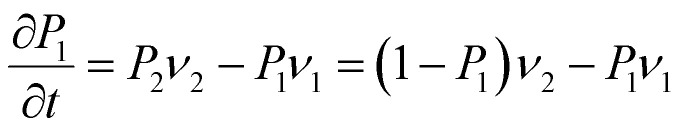


Considering ∂/∂*t* = (∂/∂*H*)(∂*H*/∂*t*) and *M* = *M*_s_(*P*_1_ cos *θ*_1_ + (1 − *P*_1_)cos *θ*_2_) eqn (7) becomes:8
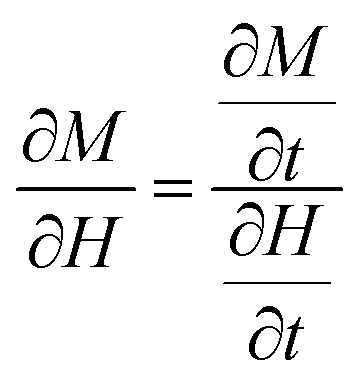
where 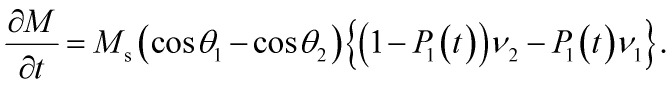


A time dependent applied magnetic field *H*(*t*) of frequency *f* with a sweeping rate 
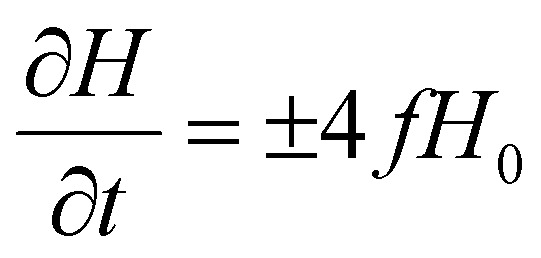
 is assumed. For negative sweeping rates, the magnetic field reversal occurs (demagnetization process). For very large values of effective anisotropy and for “easy” axis alignment along the direction of the applied magnetic field (*φ* = 0), the analytical solution of eqn (8) converges to:

9 *M* = *M*_s_ × tanh(*ξ*)

**Fig. 5:**
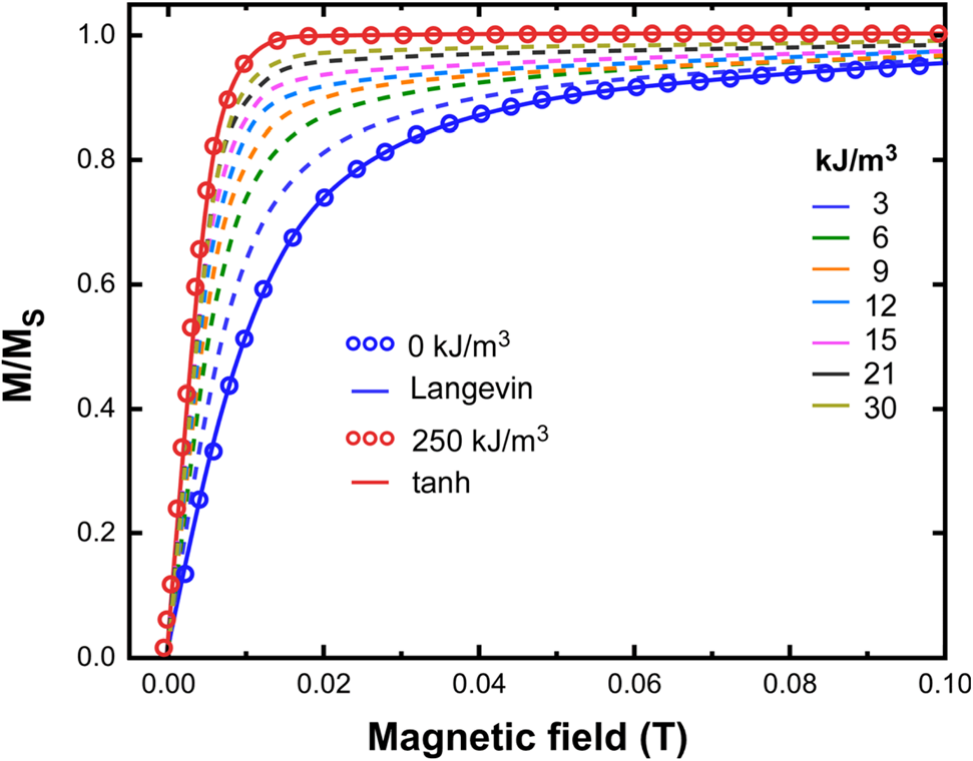
Monte Carlo simulation resulted curves of magnetization of Fe_3_O_4_ magnetic nanoparticle with diameter 40 nm *versus* external field varied from 0 to 0.1 T and for different values of the effective anisotropy. The magnetic field is applied along the easy axis (*φ* = 0).^45^

The above analysis was applied in Fe_3_O_4_ nanoparticles, used previously as a model system, to generate the virgin magnetization curve *M*(*H*). Fig. 5 depicts the calculated *M*(*H*) curves for different values of effective anisotropy. Initially, a Monte Carlo simulation is applied for various values of effective anisotropy in order to estimate *E*_1_(*K*_eff_), *E*_2_(*K*_eff_) and *E*_3_(*K*_eff_) from energy minimization. Then, for each *K*_eff_ value the corresponding (*θ*_1_, *E*_1_), (*θ*_2_, *E*_2_) and (*θ*_3_, *E*_3_) are substituted in eqn (5) and (6) to estimate *ν*_1_ and *ν*_2_, respectively. Lastly, the differential eqn (8) is solved numerically.

The solutions of this equation for different *K*_eff_ values reveal a progressive evolution of the magnetization curves from the Langevin function (0 kJ m^−3^) to the tanh function of eqn (9), (250 kJ m^−3^). When the particle's anisotropy is relatively small, the curve of magnetization follows the Langevin function where the magnetic moments can take all the possible orientations (*θ* ∈ *R*). Following effective anisotropy increment, the obtained magnetization also increases.

For large values of *K*_eff_, the magnetic moments of nanoparticles have only two stable orientations, parallel and antiparallel to nanoparticle's “easy” axis (the two minima of the energy landscape at *θ*_1_ and *θ*_2_). In the presence of magnetic field this behavior saturates faster, compared to the small anisotropy case, the magnetization and thus the Langevin function is replaced by the faster growing function tanh. Each one of the *K*_eff_ values used in simulations satisfied the prerequisite for the application of DW approximation as soon as they resulted to anisotropy fields higher than the applied magnetic field (0.1 T).

Numerical solutions of eqn (8) based on multiple demagnetization and magnetization cycles 
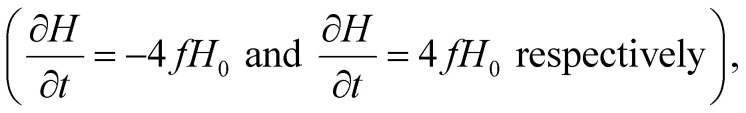
 provide the full hysteresis loop for specific values of the involved parameters (*K*_eff_, *V*, *M*_s_, *H*_0_, *f*, *T*) but only for the case of oriented particle (*φ* = 0). When the field is applied at an arbitrary angle *φ* relative to the easy axis different expressions should be given. Those expressions are crucial when modeling ensembles of non-interacting nanoparticles with random orientations. Unlike the aligned case the critical field at which the barrier vanishes depends on *φ*. The analytic expression for Δ*E*_2,1_ is more complex, but in the following we will present some useful approximations.

Starting from the original Stoner–Wohlfarth model, described by eqn (2) and holds at zero temperature, the critical (switching) field *H*_sw_ where an irreversible jump of the magnetization direction occurs, is given by the condition 
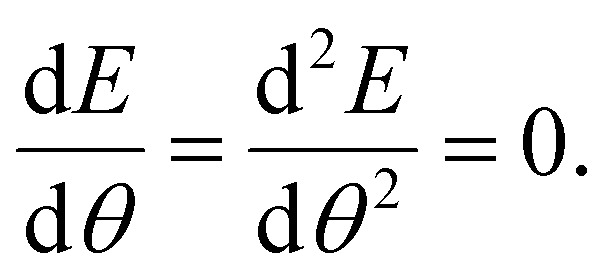
 This equations system has no algebraic closed-form for arbitrary *φ*. The explicit solution involves solving a transcendental equation, by eradicating the angle *θ*, which leads to

10 *H*_sw_ = *H*_K_[cos^2/3^ *φ* + sin^2/3^ *φ*]^−3/2^

Although there are no analytic equations, analyzing the energy difference numerically with the help of eqn (2) and (10) it turns out that the expression:11
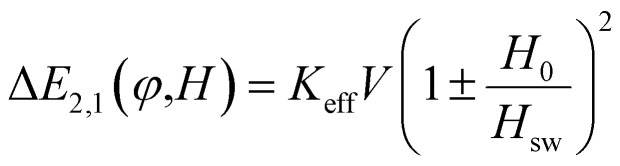
describes the results surprisingly correctly.

In the updated model at finite temperature:

• The system is assumed to be in one of two wells (*θ* = 0 or π).

• The transition rates between wells are governed by Arrhenius-type equations.

• The magnetization at time *t M*(*t*,*φ*) is a statistical average of the projection of magnetic moments in those wells onto the field direction.

The magnetization will be then given by:

12 *M*(*t*,*φ*) = *M*_s_(*P*_1_(*φ*,*t*)cos(*θ*_1_ − *φ*) + (1 − *P*_1_(*φ*,*t*))cos(*θ*_2_ − *φ*)) ⇒ *M*(*t*,*φ*) = *M*_s_(2*P*_1_(*φ*,*t*) − 1)cos *φ*

and the hysteresis loop, at the random orientation case, will occur by implementing the rate eqn (7) and (8).

To get the total magnetization *M*_total_ for a randomly oriented ensemble of non-interacting particles presenting known volume and anisotropy distributions *P*(*V*) and *P*(*K*) respectively, one has to simply integrate according to:13
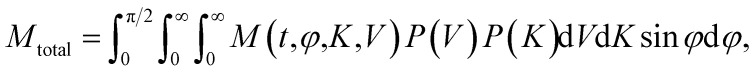
then, to substitute the eqn (12) and (13) and finally employing again the rate eqn (7) and (8).

**Fig. 6:**
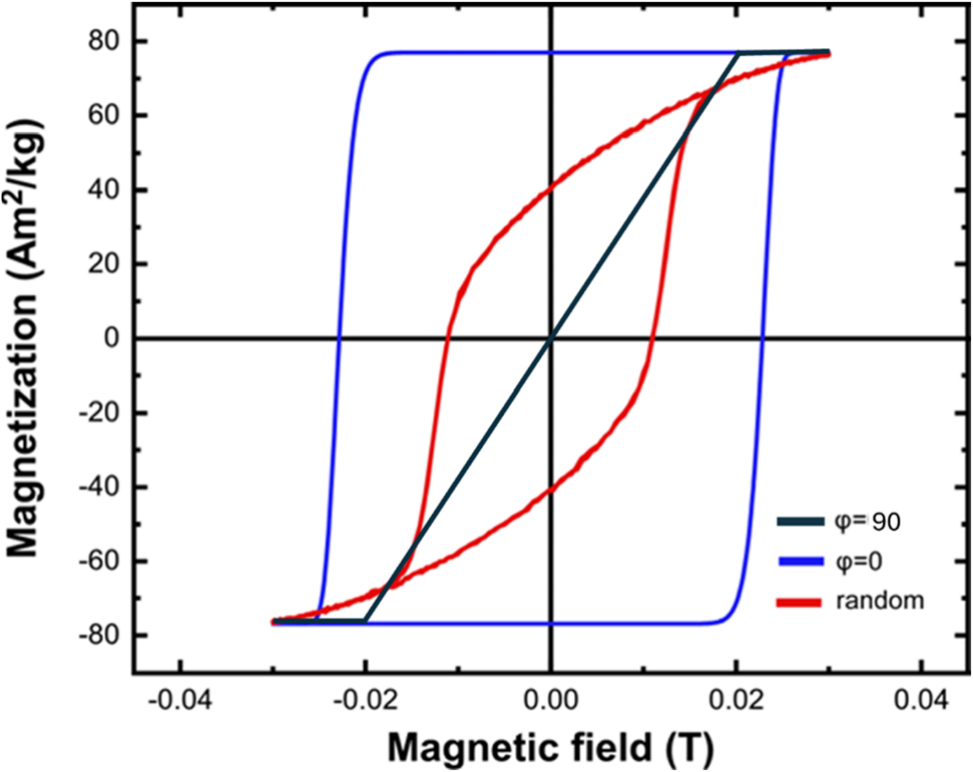
Magnetization *versus* magnetic field loops calculated when the “easy” axes are aligned with the magnetic field (blue colored loop, *φ* = 0), when easy axes are randomly oriented in space (red colored loop) and when magnetic field is applied vertically to the “easy” axis (black colored line). Hysteresis loops are obtained after solving eqn (8) for *K*_eff_ = 9 kJ m^−3^, *V* = 3 × 10^−23^ m^3^, *M*_s_ = 80 emu g^−1^, *μ*_0_*H*_0_ = 0.03 T, *f* = 300 kHz and *T* = 300 K.^45^

Both approximations (aligned and random orientation) are derived assuming thermally assisted transitions within Néel–Arrhenius-like dynamics and also that the particle does not switch deterministically, but rather probabilistically due to thermal fluctuations. This means it is especially valid when the barrier height is a few *k*_B_*T* or more: Δ*E* ≥ 10*k*_B_*T*. If the barrier is too small, thermal fluctuations cause rapid switching and the rate equation approach may no longer be meaningful. An example is presented in Fig. 6 for a spherical iron oxide nanoparticle with diameter equal to 40 nm. Suggestively, the used magnetic field amplitude and frequency are typical values of magnetic hyperthermia schemes. A simpler approach for solving the DW system is, owing to the nonlinearities of the treatment, to work for a given value of the angle *φ* and to make a numerical average over all possible *φ* values (at least, in the presence of complete randomness of the directions of the easy axes). In each case, the proposed methodology can be treated by any software, keeping in mind that the core of the script should be the numerical solution of a first-order differential equation.

The dependency of the coercive field and the hysteresis loop area to magnetic nanoparticles properties (size, anisotropy, magnetization) and applied magnetic field parameters (amplitude, frequency) is determined by fitting the numerical results to analytical equations. In the case of *φ* = 0, the following analytical expression for the coercive field is obtained:^63^14
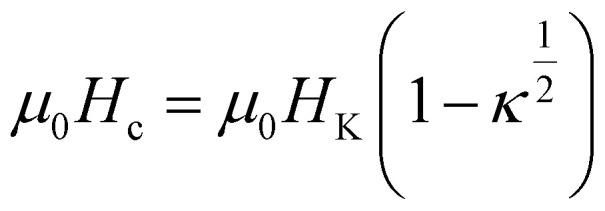
where the dimensionless parameter *κ* is defined as:15
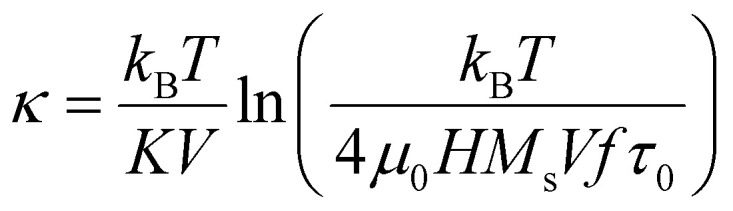


It is clear from eqn (15) that *κ* is a crucial parameter, strongly affected by the temperature, which considers the sweeping rate of the magnetic field. For the case of random magnetic field orientation, the coercive field is given by:

16 *μ*_0_*H*_c_ = 0.48*μ*_0_*H*_K_(*b* − *κ*^*n*^)

where *b* and *n* are fitting constants.

Similar to the coercive field, the estimation of the hysteresis loop area *A* is obtained by:

17 *A* = 4*a* × *μ*_0_*H*_K_(1−*κ*^1/2^)*M*_s_

when the magnetic field is aligned to the “easy” axis while for the random orientation case the followed equation is used:

18 *A* = 4*a* × 0.48*μ*_0_*H*_K_(*b*−*κ*^*n*^)*M*_s_

The key feature of hysteresis loop area formulas is the dimensionless parameter *α* which is called “squareness” and characterizes the relative area of the hysteresis loop with respect to the ideal square one. The parameter *α* is proportional to the degree of alignment. For *α* = 1 the system is perfectly optimized and the “easy” axes of all magnetic nanoparticles is aligned to the magnetic field direction. In a sense, *α* represents the degree of optimization of a given system. The maximum hysteresis area *A*_max_ that can be obtained is:

19 *A*_max_ = 4*μ*_0_*H*_max_*M*_s_

where *μ*_0_*H*_max_ is the maximum applied magnetic field. Table 1 presents the calculation of *α* from experimental results obtained on various materials of interest.

**Table 1:** Summary of experimental results from various materials of interest

System	*M*_s_ (300 K)^a^ (A m^2^ kg^−1^)	*μ*_*0*_ *H*_max_ b (mT)	*A*_max_ c (mJ g^−1^)	*A*_exp_ d (mJ g^−1^)	*α* e	Reference
Fe_*x*_O_*y*_ MNPs	≤92	13.8	5	1.5	0.3	64
Magnetosomes-A^f^	≤92	12.5	5	1.3	0.26	65
Magnetosomes-B^g^	≤92	12.5	5	2.3	0.46	65
Co MNPs	162	31.2	20.6	3.25	0.16	18
FeCo MNPs	240	29	27.8	1.5	0.054	66
Fe MNPs	218	66	57.5	5.6	0.097	67
CoFe_2_O_4_	75	31.1	9.35	0.63	0.067	68

a Bulk magnetization per unit mass at 300 K.

b *μ*_0_ *H*_max_ magnetic field of experiments.

c *A*_max_ theoretical maximum hysteresis area that could have been measured, which was calculated using eqn (19).

d *A*_exp_ hysteresis area experimentally measured under these conditions.

e Calculated as *α* = *A*_exp_/*A*_max_.

f Randomly-oriented iron oxide nanoparticles synthesized by bacteria.

g Iron oxide nanoparticles synthesized by bacteria and aligned with the magnetic field.

## Many-particles approximations: numerical models in MPH

Understanding MNPs magnetization dynamics is crucial for optimizing their heating efficiency. Due to the complex nature of inhomogeneous magnetization in nano-systems, the use of dynamical micromagnetic approaches is necessary. In this regard, full-scale numerical micromagnetic simulations have become essential for accurately modeling the response of a large number of MNPs under AMF. Over the past decade, advancements in computational power and the development of specialized micromagnetic software packages—such as OOMMF, LLG, MicroMagus, MuMax, and NMAG—have enabled detailed investigations into the magnetization dynamics of nanoparticles in MPH (Dmytriiev *et al.* 2012; Leliaert *et al.* 2018; Leliaert and Mulkers 2019; Stavrou *et al.* 2019; Sundara Mahalingam *et al.* 2019). Micromagnetic modeling in MPH is based on Brown's equation, which defines the total energy of a ferromagnet, and the Landau–Lifshitz–Gilbert (LLG) equation, which governs the magnetization dynamics under an external field. By solving these equations numerically, researchers can predict hysteresis losses which are critical for designing MNPs with optimized heating performance for hyperthermia applications.

Under the assumption of micromagnetic theory, Brown^69^ derived a set of equations after employing the magnetic Gibbs free energy, such as:

20 *E* = ∫(*E*_ex_ + *E*_a_ + *E*_z_ + *E*_d_ + *E*_dip_)

This integral in eqn (20) runs over the total volume of the ferromagnetic body. The details of constituent energies are mathematically described by eqn (21)–(25) and are theoretically defined as follows: exchange energy *E*_ex_ (eqn (21)) indicates the interaction of spins with nearest neighbors. Volume anisotropy energy *E*_a_ (eqn (22)) indicates the crystal structure and depends on the crystal type of specimen material *i.e.* uniaxial or cubic symmetry. The Zeeman energy *E*_z_ (eqn (23)) indicates an externally applied magnetic field *H*. The term *E*_d_ indicates the dipolar nature of the individual magnetic nanoparticles that produce a demagnetizing field, also called the stray field, and triggers a demagnetizing energy contribution (eqn (24)), while *E*_dip_ refers to the magneto-dipolar interaction between moments (eqn (25)). In the case of an assembly of *N* magnetic nanoparticles, the energies involved are given by the following expressions:21
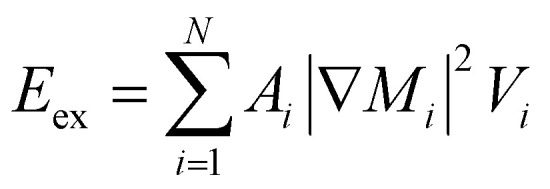
22
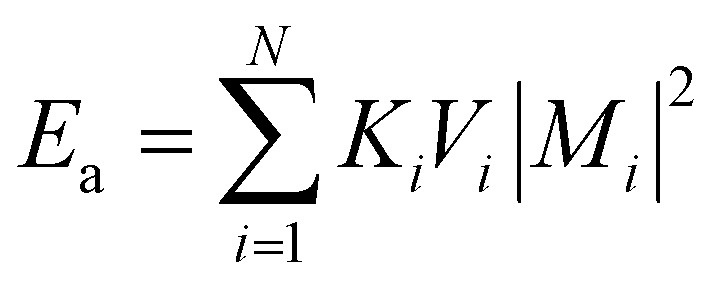
23
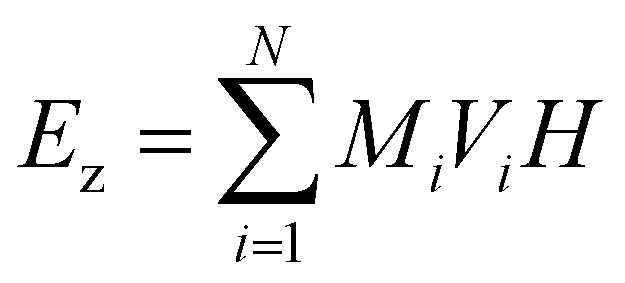
24
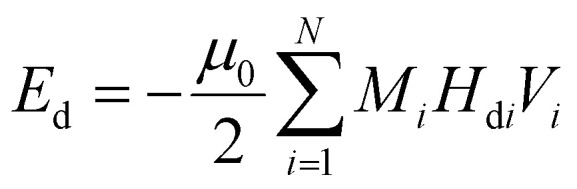
25
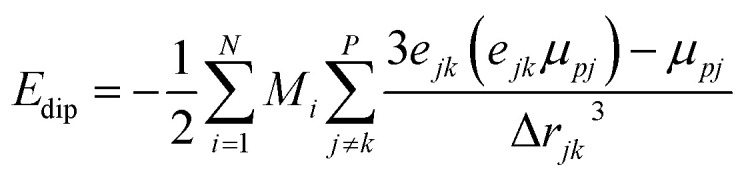
where *A*_*i*_ is the exchange-stiffness constant, *M*_*i*_ the magnetization vector and *H*_d*i*_ the demagnetizing field of the *i*-th particle. In eqn (25), *μ*_*pj*_ is the magnetic moment vector of the *j*-th particle and Δ*r*_*jk*_ the distance between *j*-th and *k*-th particles. Magnetic moment vectors of particles *m*_*p*_ are treated as point dipoles located in the centers of closely generated packed spheres while at the macroscale level *M*_*i*_ is the total magnetization of individual particles.

Eqn (20) generates an effective magnetic field *H*_eff_ which accounts for all relevant contributions to the magnetic Gibbs free energy *E* such as the externally applied magnetic field *H*, the demagnetizing field *H*_d*i*_, the dipolar magnetic field and the exchange field. When a ferromagnetic material is placed in a magnetic field it's magnetization vector *M*_*i*_ “moves” due to the influence of *H*_eff_. This motion is well described by the Landau–Lifshitz theorem^70^ which is expressed by the following equation:26
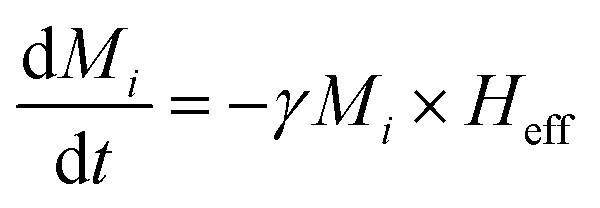


This equation describes the precession of magnetization vector *M*_*i*_ in an effective field *H*_eff_. On the other hand, hysteresis curves tell us that beyond a certain value of an applied magnetic field, any magnetic sample can become saturated, *i.e.*, all moments in the material are aligned along the field direction and thus, precession alone cannot describe this process. Energy dissipation (or damping) must be included to allow for magnetization to relax toward the saturated state. This is the reason why Gilbert^71^ included a phenomenological damping parameter in eqn (26) to express the experimentally noticeable damping in ferromagnetic materials. Consequently, the LLG equation is given by:27
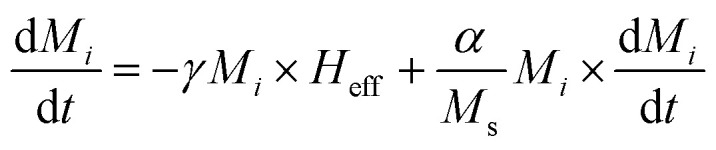
where *α* is the damping parameter and *γ* is the gyromagnetic ratio. The resulting dynamics from eqn (27) is a damped processional motion of magnetization vector *M*_*i*_, of each magnetic nanocomposite phase, around the effective field.

Moreover, thermal field is added so the effects of finite temperature on magnetisation are considered. This is done by including a random noise field.^72^ The magnitude of the noise field is assumed to be the same in all three directions (isotropic), with a zero mean. The subsequent thermal fluctuations are also assumed to be uncorrelated. In other words, this assumption amounts to presuming the thermal noise to be white, with a flat power distribution in all frequencies. Mathematically, these assumptions can be written as follows: 〈*H*_*th*_〉 = 0, 
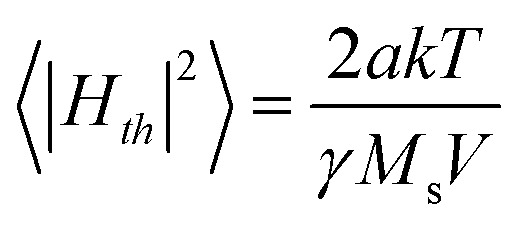
 and 〈*H*_*th*_(*t*)*H*_*th*_(*t* + *τ*)〉 = |*H*_*th*_|^2^*δ*(*τ*). An important point to stress is the time-dependence of the noise field: the exact magnitude of the noise field depends on the frequency of observation, and therefore the selection of the time-step in the discrete simulation is critical.

**Fig. 7:**
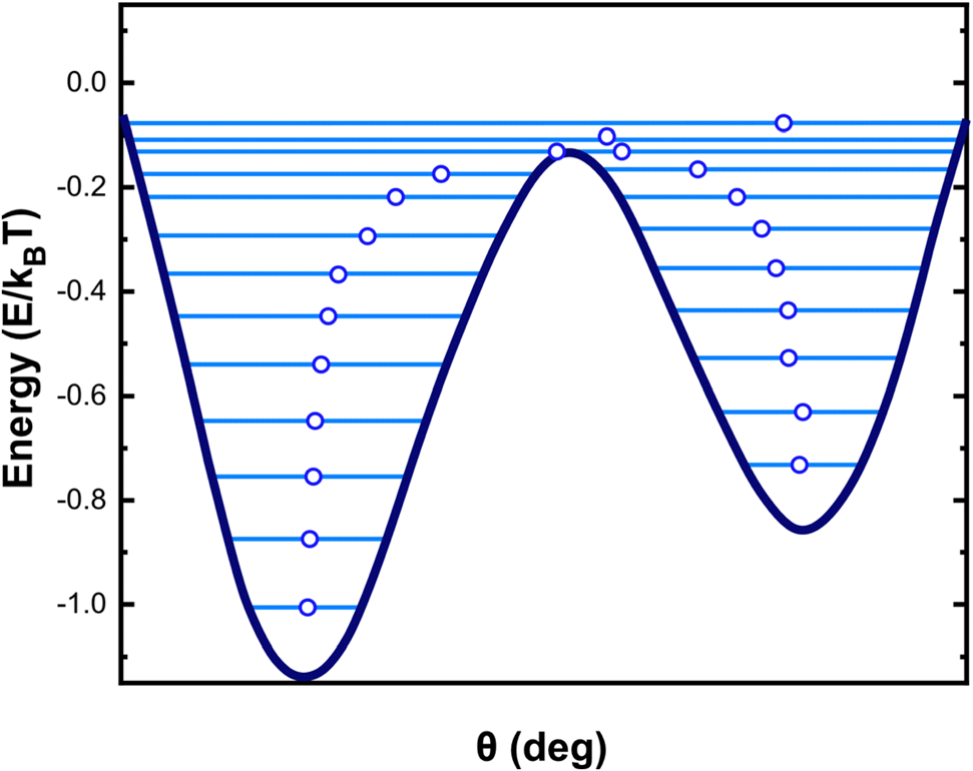
Schematic diagram of additional energy states emerged for a DW magnetic nanoparticle when thermal field is activated in the micromagnetic approach.^45^

When thermal agitation is active, the system has a chance to overcome the barrier before its height is reduced to zero and the jump has some probability of taking place at an earlier time.^73^ In addition, the chances for this to occur should be higher the lower the field rate of change, because the system spends more time in front of the barrier to overcome, a process that is also imposed by the magnitude of magnetic anisotropy.^3^ As shown in Fig. 7, additional energy states are raised due to the thermal interactions between particles defining an inhomogeneously magnetized material. Nevertheless, in literature, the hysteresis loop is considered independent of the field rate. Thus, the estimation of specific absorption rate values (SAR), a key measure used for characterizing the heating efficiency of nanoparticles in MPH, from hysteresis loop area A, leads to a direct proportionality between SAR and frequency *f*, revealed through the linear relationship: SAR = *A* × *f*. However, this relationship is far from true since, as we explained rate-independent hysteresis is just a zero-temperature approximation, and so *A* should be described as a function of frequency *A*(*f*) at higher temperatures and for low frequencies.

**Fig. 8:**
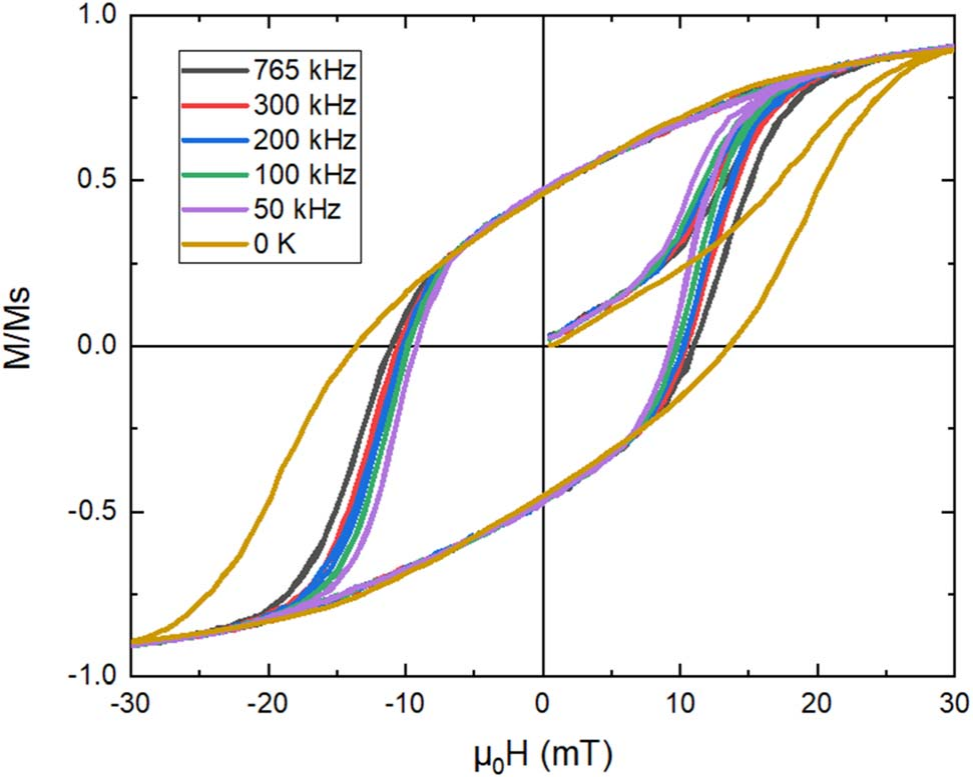
AC hysteresis loops for 30 mT magnetic field amplitude and for various frequencies (50–765 kHz). The largest loop corresponds to 0 K and is independent of the frequency.^35^

In^74^ the dynamic hysteresis loops are derived from micromagnetic simulations for the various frequency values and are depicted in Fig. 8 together with the loop obtained at 0 K which is independent on frequency. From the results is shown that there are two frequency regimes. One low frequency regime until ≈400 kHz where the hysteresis area increases with frequency due to the increase of the phase delay in the magnetization response. After this critical value of frequency, MNPs magnetization reaches the maximum phase delay *i.e.* the maximum relaxation time of spins. The whole process is dictated by thermal phenomena. At low frequency values thermal fluctuations dominate and, thus, the magnetic moments of MNPs need more time to surpass the anisotropy barrier. On the other hand, at room temperature and in the presence of an AMF, magnetic moments will overcome the barrier before its height is decreased and the jump has some finite probability of happening before AMF amplitude reach to zero. The increase in frequency diminishes the temperature influence and induces a kind of small hardening to the MNPs loop.

**Fig. 9:**
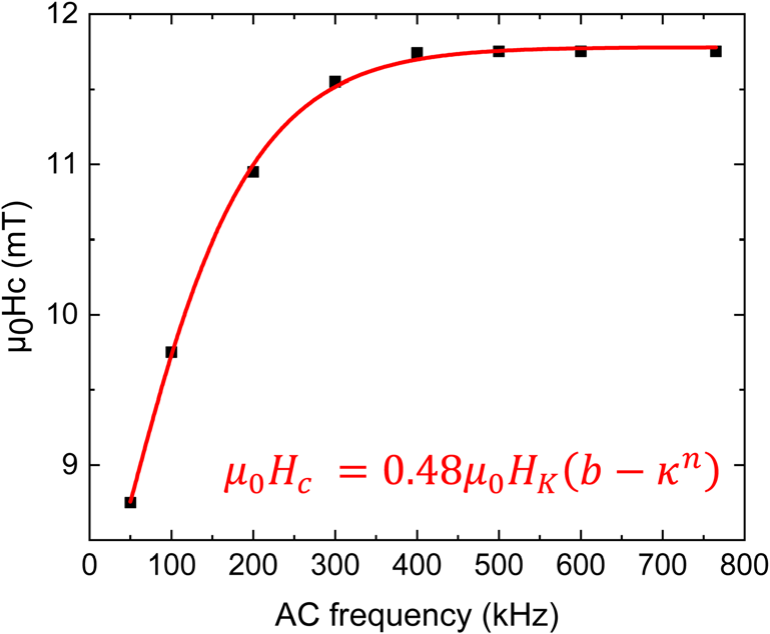
Dependence of the hysteresis loop with frequency and fitting of obtained data. The fitting curve and the corresponding equation are shown with red color.^35^

This behaviour is also illustrated in Fig. 9 through the dependence of the coercive field *μ*_0_*H*_c_ with frequency, where *H*_c_ is the magnitude of the reverse magnetic field strength. The results were also fitted with eqn (16).

**Fig. 10:**
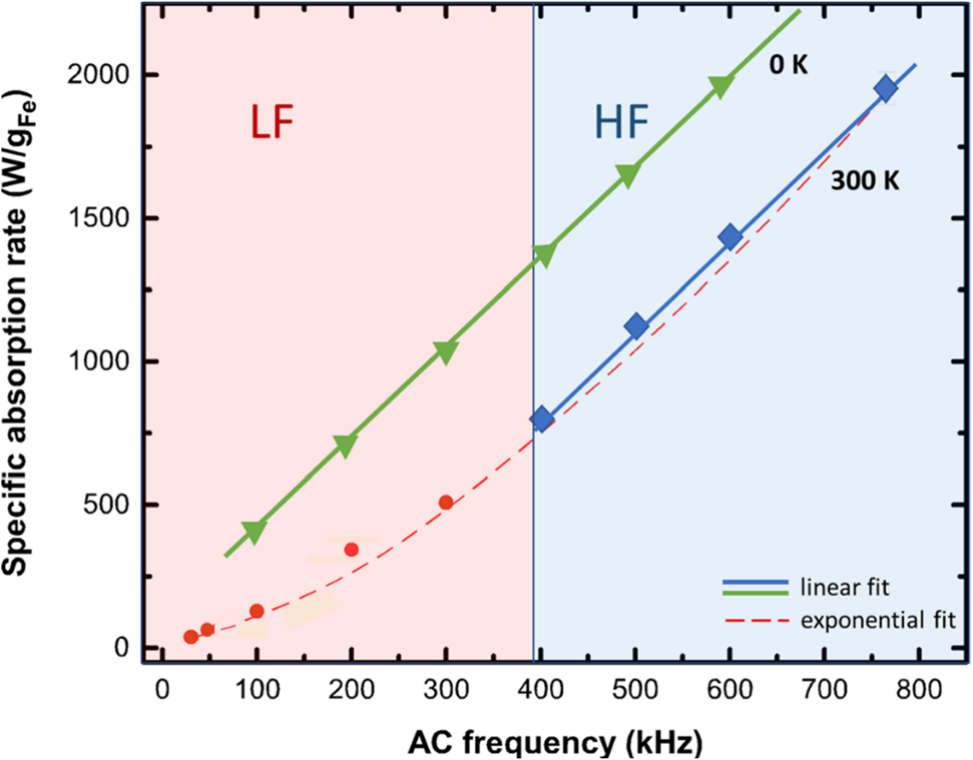
SAR dependence on frequency for 0 and 300 K. At the low frequencies (LF) regime and room temperature, the hysteresis loop area is not constant with frequency and thus SAR(*f*) diverges from linearity. An exponential trend is observed until the critical frequency value of 400 kHz. Above this value, the linear trend is restored at the high frequency (HF) regime. At 0 K a rate-independent loop results to a linear behavior of the SAR(*f*) function in both regimes. Note here that SAR is estimated in watts per iron mass which is the percentage of iron in the magnetic nanoparticles mass. The system under study were an assembly of 40 nm magnetite nanoparticles exposed to an AMF with an amplitude equal to 30 mT.^74^

The SAR dependence on frequency is obtained by employing the equation SAR = *μ*_0_∮*M*(*H*)d*H* × *f* where the integral gives the hysteresis loop area. The results for 0 and 300 K are presented in Fig. 10.

In the LF regime the exponential fitting is given by: SAR = *S*_0_ × *e*^*ηf*^ where *S*_0_ was found equal to 70 W g_Fe_^−1^ and corresponds to the hysteresis losses of the static (zero frequency) loop and *η* is a fitting constant. Thus, in the LF regime the SAR (*f*) relationship is more suitably described by an exponential trend rather than a linear relationship usually employed in literature, and is valid only in the HF regime.

**Fig. 11:**
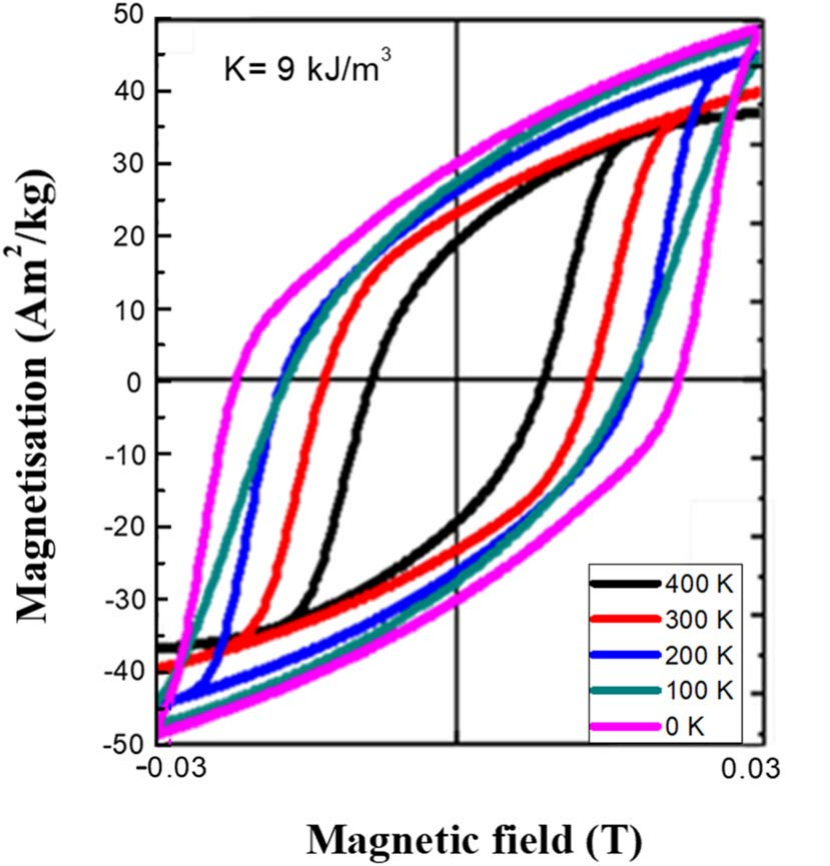
Dynamic hysteresis loops of single domain magnetite MNPs estimated by micromagnetic simulations at various temperatures and compared to the one observed at 0 K. The applied field amplitude and frequency were equal to 30 mT and 765 kHz respectively. The value of anisotropy constant was equal to 9 kJ m^−3^, a value also used in analytical models as depicted in Fig. 4 for magnetite. The decrease of hysteresis loop area and thus the decrease of hysteresis losses is obvious with temperature increase.

To illustrate the temperature effect on the hysteresis loop we show in Fig. 11 some *M*(*H*) curves for various temperatures from 0 K to 400 K with a step of 100 K and magnetic field variations from −30 mT to 30 mT as above. The value of frequency is now fixed at the value of 765 kHz which is a typical value used in MPH. The *M*(*H*) curves, obtained by micromagnetic simulations, in Fig. 11 illustrate the influence of the thermal fluctuations on the hysteresis curves. For low-temperatures case, the system is in the blocked state and exhibits ferromagnetic-like hysteresis losses that originate from the overcoming of the anisotropy energy barrier while for the high temperatures thermal excitations are large enough to promote the reversible jumping over the anisotropy barrier without energy losses. The magnetic moments fluctuate because of the thermal energy and consequently fluctuations are reduced the smaller the temperature. Thus, the physical tendency of both saturation magnetization and coercive field decrease with temperature increase is observed from the simulations and coincides with theory. Magnetization saturation and coercive field values are depicted in Table 2.

**Table 2:** Summary of numerical results on magnetic properties as a function of temperature

*T* (K)	*M*_s_ (A m^2^ kg^−1^)	*μ*_0_ *H*_c_ (mT)
0	50	25
100	48	22
200	45	21
300	40	18
400	34	12

Another interesting plot that may be followed from the temperature dependent hysteresis loop curves is the one for the variation of saturation magnetization with temperature. The temperature dependence of the magnetization, investigated by using a combination of different experimental methods, is an important source of information regarding the anomalies and/or singularities linked to the dimensional confinement in magnetic nanoparticles. The deviation shown by magnetic material if compared with its highest magnetization state is given by the spin-wave excitation, also called magnon. The latter is basically originated by the sinusoidal-type distribution of the spin orientation states within the material, which are forming a certain angle with respect to each other; thus, a more energetically unfavorable situation of anti-parallel coupling is avoided. Departing from the spin-wave theory, Bloch proposed an expression^75^ aiming to describe the thermal dependence of the saturation magnetization *M*_s_(*T*) in a bulk material,28
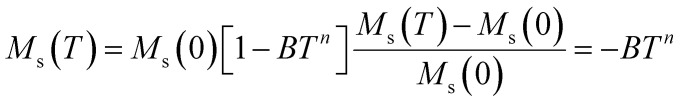
where *M*_s_(0) is the saturation magnetization at 0 K, *n* is the Bloch exponent—originally equals to 3/2—and *B* is a constant that depends on the spin-wave stiffness and, thus, on the inverse of the exchange integral *J* in the following way 
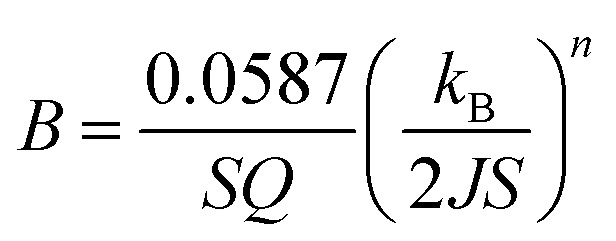
 and measured in K^*−n*^ where *Q* is 1, 2, and 4 for single cubic, bcc, and fcc systems, respectively, and *S* is the total electron spin. The exponent *n* = 3/2 fits well to experimental results in ferromagnetic materials and some spinel ferrites such as Mn_*x*_Fe_3−*x*_O_4_ (0.2 ≤ *x* ≤ 2).^76^

**Fig. 12:**
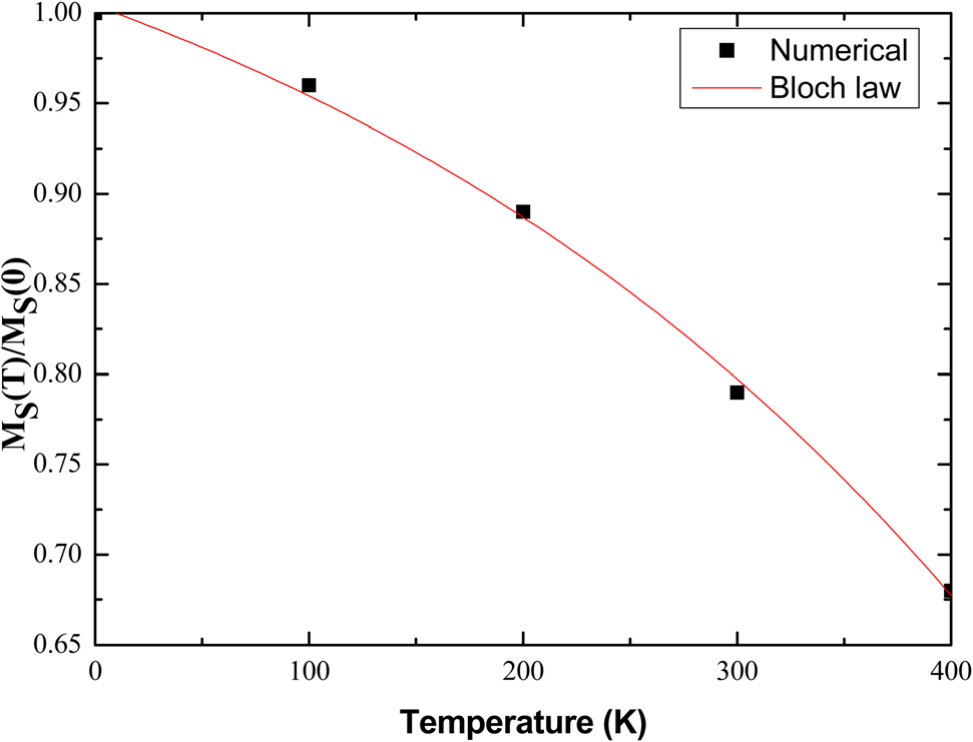
Saturation magnetization normalized to saturation magnetization at 0 K *vs.* temperature at various temperatures obtained by extracting magnetisation data from the temperature dependent hysteresis loops resulted from our implemented micromagnetic simulations. The continuous red line represents the fitting of the *T*^*n*^ Bloch law. The exponent *n* value that best fits our numerical data is equal to 2 while the Bloch constant *B* is found equal to 3.03 × 10^−6^ K^−2^.

By taking the saturation magnetization data that occurred from Fig. 11 and analyze them using Bloch's law and determine the Bloch's constant and exponent, *B* and *n* respectively, directly from the fitting (*B* and *n* are left as free-fitting parameters) of the saturation magnetization to temperature plot with eqn (28) as illustrated in Fig. 12.

The Bloch's law, given by eqn (28), indicates that the decrease in the saturation magnetization with increasing temperature due to spin-wave excitations is described by a power law in *T*. Our data seem to follow the predicted behavior as displayed in the plot of Fig. 12. There, the solid red line represent the fit of the eqn (28) and the agreement between the latter and numerical data is optimized (*R*^2^ = 0.995) for *n* = 2 a value that deviates from Bloch's law *n* = 3/2. In the case of nanoparticles and clusters, some theoretical calculations have shown that the exponent *n* is higher than 3/2, and reaches 2 as a consequence of the reduction in the size of the particles.^75^ Going back to eqn (28), such deviations are numerically reflected in the temperature exponent and the *B* constant value, which are increased with respect to the bulk values, indicating a decrease in the Curie temperature of the studied system. The main idea behind the explanation of this effect is that the lack of full coordination at the surface of the MNPs may lead to larger spin deviations in this region than in the central part of the MNP. The effect of the limited number of degrees of freedom at the surface leads to an energy gap in the spin-wave spectrum resulting in a flat magnetization curve at low temperatures. In other words, the magnetization decreases faster at higher temperatures in the nanoparticles than in the bulk material, due to lacking coordination at the surface. Both micromagnetic calculations^45,77^ and experimental results^78^ have repeatedly shown this behavior. Bloch's constant *B* is also left as a free-fitting parameter, determined from the fitting procedure and found equal to 3.03 × 10^−6^ K^−2^ a value that has been already reported in the literature for single domain ferromagnetic nanoparticles.^79^

The explained methodology can be extended to optimize the structural and magnetic properties of magnetic nanoparticles used in MPH (crystal symmetry, grain size, anisotropy, saturation magnetization, coercive field).^36,72,77,80–82^ Since nanoparticles synthesis, characterization and evaluation procedures can be quite costly and time-consuming, the use of numerical simulations instead of experimental measurements to rapidly and accurately examine a large number of parameters and properties and finally, obtain the optimum ones, comes as an advantageous opportunity. Offered possibilities of employing modern numerical methods and suitable computer software are in position to easily deliver a reliable prediction on the hysteresis behavior of any system designed to operate as an MPH agent.

## Discussion on rate equations and numerical models

In the study of magnetic hyperthermia, understanding and predicting the behavior of magnetic nanoparticles under alternating magnetic fields is crucial for optimizing heating efficiency. Researchers employ various modeling approaches to describe the dynamic magnetization processes, ranging from full-scale numerical simulations based on the Landau–Lifshitz–Gilbert (LLG) equation to analytical approximations like the rate equation model under the double-well potential framework. While both approaches provide valuable insights, analytical models offer several distinct advantages when dealing with the physical realities of hyperthermia experiments, especially considering the immense number of particles involved.

In a typical magnetic hyperthermia experiment, the sample contains a massive number of nanoparticles. For instance, in one milligram of MNPs, the number of individual particles can exceed 10^13^, assuming a typical particle mass on the order of 10^−22^ kg. The total number of particles per unit volume can be estimated by dividing the sample's saturation magnetization (*e.g.*, measured in A m^−1^) by the magnetic moment of a single particle (A m^2^). This huge number of particles underscores a fundamental limitation of direct numerical simulations: it is computationally infeasible to model each particle individually. Numerical simulations using the LLG equation (implemented in software like OOMMF or MuMax3) solve the time evolution of the magnetization vector for individual or assemblies of magnetic moments under the influence of external and internal magnetic fields. These methods are highly accurate and account for detailed micromagnetic interactions, including exchange coupling, dipole–dipole interactions, thermal noise, and anisotropy effects. However, this accuracy comes with significant computational cost:

(1) Particle scale modeling LLG simulations are generally restricted to simulating a few particles or a small grid of magnetic cells. Simulating 10^13^ particles, as in a real hyperthermia sample, is completely infeasible due to limitations in memory and processing power.

(2) Statistical limitations: since LLG simulations model only a tiny fraction of the actual system, they may not accurately capture the collective response of an ensemble with distributed particle sizes, anisotropies, or orientations.

(3) Neglect of thermal fluctuations or approximations: including thermal effects requires stochastic LLG formulations, which significantly increase computational complexity and often require simplifications that reduce realism.

(4) No explicit particle count: these simulations often model a continuous magnetization distribution rather than discrete particles. As such, they cannot explicitly incorporate the actual number of particles, limiting their usefulness in estimating macroscopic quantities like specific absorption rate (SAR) per unit volume.

In the double-well approximation, the time-dependent magnetization *M*(*t*) is obtained by solving the rate equation. This approach offers several key advantages:

(1) Scalability: since each particle is treated independently (under the assumption of negligible interparticle interactions), the model can be scaled to an arbitrary number of particles. One can directly calculate the macroscopic magnetization by summing or integrating over the contributions of all particles, weighted by distributions in size, anisotropy, and orientation.

(2) Direct incorporation of particle number: unlike LLG models, analytical models can explicitly include the number of particles per unit volume. This allows direct estimation of heating power or SAR by calculating:SAR = *N*_s_*μ*_0_∮*M*(*H*)d*H* × *f*where *N*_s_ is the number of particles per sample mass in kg^−1^. This feature is essential for linking theoretical models with experimental measurements.

(3) Inclusion of distributions: analytical models can easily accommodate realistic distributions of particle properties (*e.g.*, log-normal size distribution, random orientation of easy axes, or Gaussian anisotropy distribution). These distributions are integrated into the final magnetization response using statistical mechanics tools, something that becomes highly complex in LLG simulations.

(4) Faster computation: analytical solutions or numerical integration of rate equations is many orders of magnitude faster than LLG-based simulations, making it feasible to explore wide parameter spaces (*e.g.*, different field amplitudes, frequencies, temperatures) efficiently.

(5) Thermal effects naturally included: since the flipping rates are based on thermal activation over energy barriers, the role of temperature is naturally incorporated without the need for stochastic differential equations.

Analytical models are rooted in equilibrium and nonequilibrium statistical mechanics. The double-well system is a prototypical example of a bistable system in statistical physics, where the populations of each state evolve with time according to thermally activated transition rates. This connection allows for a principled approach to modeling macroscopic observables like net magnetization, entropy production, or hysteresis losses, using well-established tools. Averaging over distributions is done through integrals of the form presented in eqn (13). Such ensemble averaging is essential for comparison with experiments where particle heterogeneity is the norm.

On the other hand, LLG simulations provide microscopic detail and are valuable for understanding local magnetization dynamics and complex particles interactions such as the magnetostatic and dipolar interaction leading to strong demagnetizing fields. Moreover, the main advantages are:

• It captures full vector dynamics including precession, damping, and transient behavior.

• Works well at very high frequencies or short timescales.

• Necessary when anisotropy is complex or field varies non-sinusoidally.

If researchers working in this field aim in understanding and optimizing magnetic hyperthermia heating efficiency, rate equations methods are typically preferable, unless they study effects that require full LLG dynamics such as high-speed switching, precession-dominated regimes and most importantly strong interactions between nanoparticles.

## Linear response theory

In MPH, the linear response theory (LRT) is a model that aims to describe the dynamic response of an assembly of small (<20 nm) superparamagnetic nanoparticles using the Néel–Brown relaxation time, a concept that will be further analyzed below. The starting assumption of LRT is that the magnetic system responds linearly with the magnetic field and its magnetization can be put in the form *M*(*t*) = *χH*(*t*) where *χ* is the complex susceptibility.^83^ When an alternating magnetic field of sufficiently high frequency is applied, the magnetisation of a superparamagnetic particle lags behind the applied field. As a result of this phase lag, the susceptibility, *χ* = *χ*′ − *iχ*′′ is an imaginary quantity with the real part *χ*′ representing the in-phase component, and the imaginary part, *χ*′′ the quadrature or loss-component are given by 
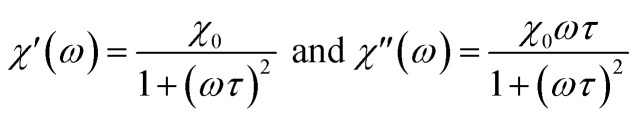
 where *χ*_0_ is the equilibrium magnetic susceptibility. The equilibrium susceptibility depends on the applied magnetic field.^75,84,85^ A conservative approach consists in treating *χ*_0_ as the chord susceptibility represented by a Langevin function *L*(*ξ*) = coth*ξ* − 1/*ξ* implemented in the following expression: 
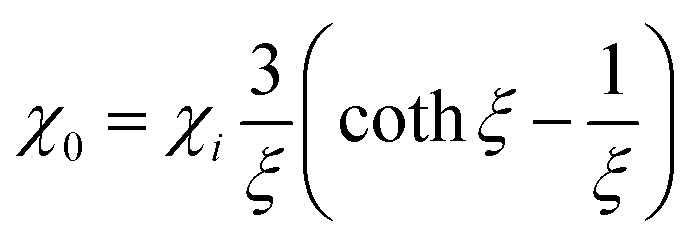
 where 
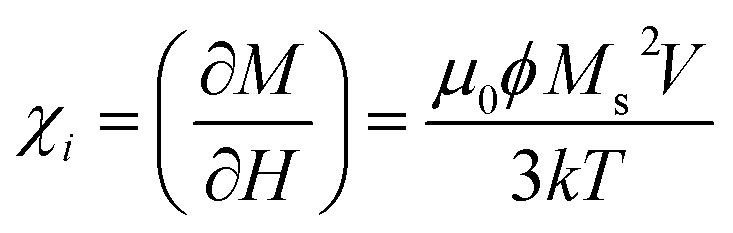
 is the initial susceptibility and *ϕ* the fraction of saturation magnetisation *M*_s_ of the magnetic nanoparticles to the bulk magnetisation *M*_d_: *φ* = *M*_s_/*M*_*d*_. Although *χ*′ decreases with increasing frequency, the imaginary part, *χ*′′, peaks at an angular frequency 
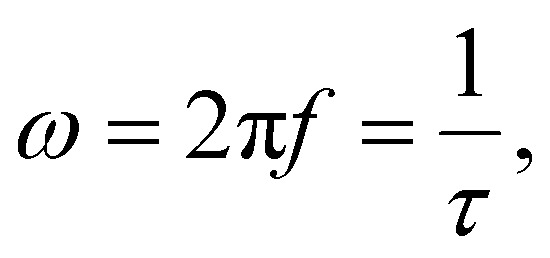
 where *τ* is the relaxation time of the particles. These relationships are identical to the Debye spectra of polar molecules in the absence of a constant field. Note here that, ferromagnets exhibit magnetic resonance at frequencies ≈ 10^8^ to 10^10^ Hz yielding a change of sign to negative value of *χ*′(*ω*) and a sharp peak of *χ*′′(*ω*).^86,87^

When an external magnetic field that provides sufficient energy is applied to a system of superparamagnetic nanoparticles suspended in a liquid carrier, the magnetic moment of the latter may be displaced from its preferred orientation. The whole system of the liquid and the magnetic nanoparticles is called magnetic fluid or “ferrofluid’’.^88^ Consequently, as the magnetic moment returns to the equilibrium state, thermal energy is released. There are two possible relaxation mechanisms of the magnetic moment, Néel and Brownian.^89^ In Néel relaxation which is associated with the anisotropy energy, the magnetic moment alternates between parallel and anti-parallel directions within the MNPs, while the physical orientation of the particle remains fixed. In the Brownian relaxation mechanism, which relates to the hydrodynamic properties of the magnetic fluid, the physical orientation of the particle changes, while the magnetic moment remains unaltered with respect to the particle rotation axis. In the Néel mechanism, the relaxation time, *τ*_N_, is given by:^63^29
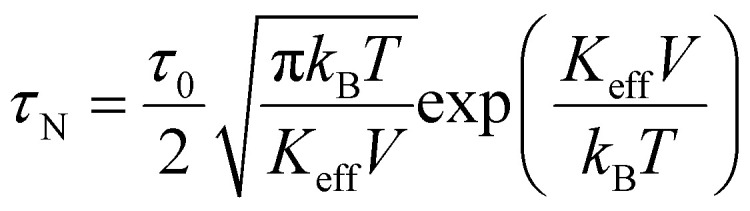
while Brownian mechanism the relaxation time *τ*_B_ is given by30
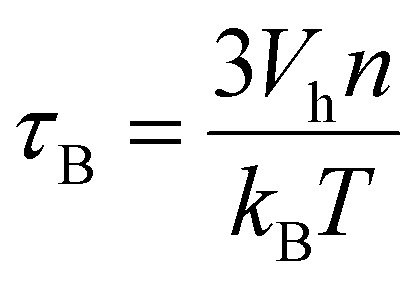
where *V*_h_ denotes the hydrodynamic volume obtained from the hydrodynamic diameter measured, for example, through dynamic light scattering and *n* the viscosity of the liquid carrier. The hydrodynamic volume of a monodispersed nanoparticle is given by the relationship 
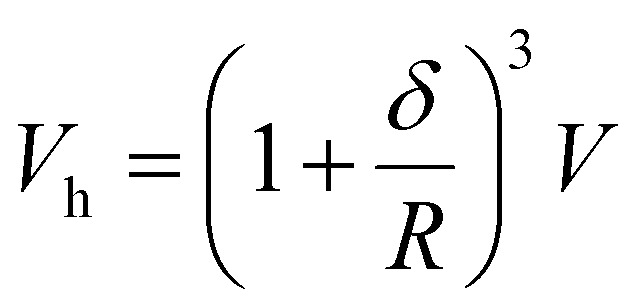
 where *R* is the nanoparticle radius and *δ* is the thickness of the surfactant. Néel relaxation dominates over a short range of particle sizes as the fastest process. Above a certain critical size, Brown relaxation mechanism, depicted by eqn (30) takes over. In the region of that critical size, where both processes are equally probable, the net relaxation time *τ* is given by their geometric mean:^37^31
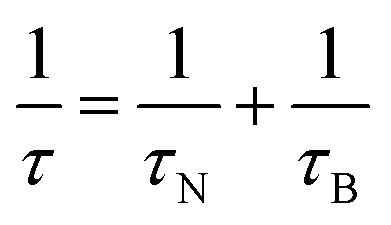


The energy deposition premises an AMF with reversal times comparable to the effective relaxation time. Then, thermal energy is deposited due to delay in the relaxation of magnetic moment. Although both mechanisms are present in the heating process, a transition between both mechanisms occurs when *τ*_B_ = *τ*_N_ and depends on several parameters including the crystal and hydrodynamic volume of the MNPs, the frequency of the AC field, the energy barrier and the viscosity of the medium. Specifically, Néel relaxation mechanism is dominant for smaller particles of low anisotropy, suspended in viscous medium, at high frequencies of the AMF, while Brownian relaxation mechanism is favored for larger particle sizes with high anisotropy, in a medium of low viscosity and at low AMF frequencies. When the MNPs are immobilized in a medium, for instance in tumor tissue, or form agglomerations, Brownian relaxation mechanism is suppressed and Néel relaxation is the only occurring mechanism.^90^ Exploitation of magnetic particles that deposit energy through Néel relaxation mechanism is encouraged in medical applications, since Brownian relaxation mechanism is influenced by the local environment, which introduces further implications in treatment planning.^10,85,91–94^

The computation of power dissipation in a magnetic fluid due to susceptibility losses is accomplished by usage of thermodynamics theory. Rosensweig developed a model that calculates the volumetric power dissipation rate through analytical relationships.^13,89^ The model applies at low fields, hence within the linear response regime of magnetization. According to the first law of thermodynamics, the internal energy *U* for a system of fixed density can be expressed as the sum of the heat *Q* added to the system and the magnetic work *W* applied on the system. For a unit volume:d*U* = *δQ* + *δW*.

Assuming an adiabatic process *δQ* = 0 and the differential internal energy of the system equals to the differential magnetic work

32 d*U* = *δW* = *H* × d*B*

where *B* the induced magnetic field and *H* the magnetic field intensity within the sample. Due to collinearity of the fields the relationship can be written in terms of magnitudes as d*U* = *H*d*B* with *B* = *μ*_0_(*H* + *M*) and by substitution in eqn (32)

33 Δ*U* = −*μ*_0_∮*M*d*H*.

The magnetization *M* of the magnetic fluid can be expressed in terms of the complex magnetic susceptibility *χ* = *χ*′ − *iχ*′′. The external magnetic field *H*(*t*) and resulting magnetization *M*(*t*) are given by:

34 *H*(*t*) = *H*_0_ cos *ωt* = Re[*H*_0_e^*iωt*^] and *M*(*t*) = Re[*χH*_0_e^*iωt*^] = *H*_0_(*χ*′ cos *ωt* + *χ*′′ sin *ωt*)

respectively. From eqn (34) it is obvious that *χ*′ is the in-phase component and *χ*′′ the out-of-phase component of *χ*. By substitution of *M*(*t*) and *H*(*t*) from eqn (34) in eqn (33) the following expression for internal energy is obtained:35
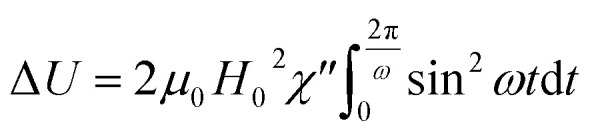


Only the *χ*′′ component of magnetic susceptibility also referred as the loss component is present in eqn (35). Thus, the time dependent magnetization will be given by the equation:^95^36
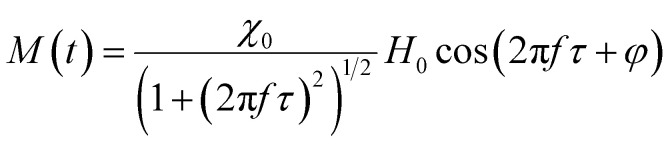
here *φ* is the phase difference between the two signals *H*(*t*) and *M*(*t*) and is given by *φ* = arctan(2π*fτ*).

It has been demonstrated by Rosensweig that the volumetric power dissipation is then given by the product of the internal energy Δ*U* and cyclic frequency *f* = *ω*/2π:

37 *P* = *f*Δ*U* = *μ*_0_π*χ*′′*fH*_0_^2^.

if we substitute the *χ*′′ relationship 
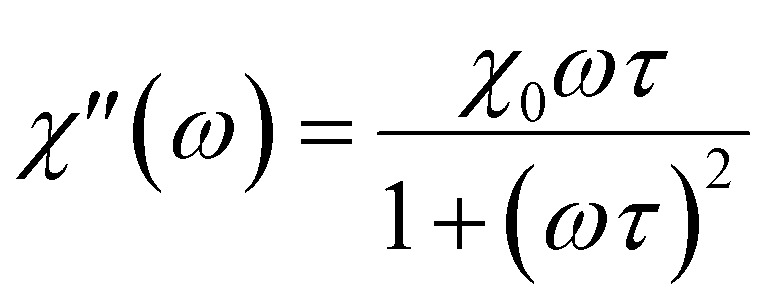
 in eqn (37) yields the following expression for the volumetric power dissipation corresponding to an aqueous monodispersion assembly of magnetic nanoparticles:38
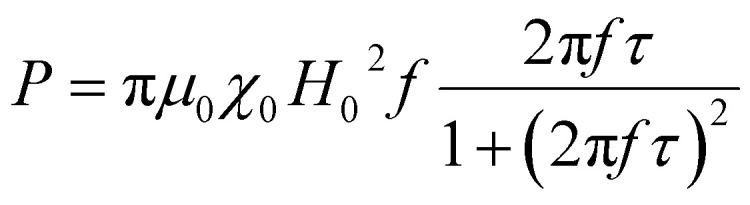


**Fig. 13:**
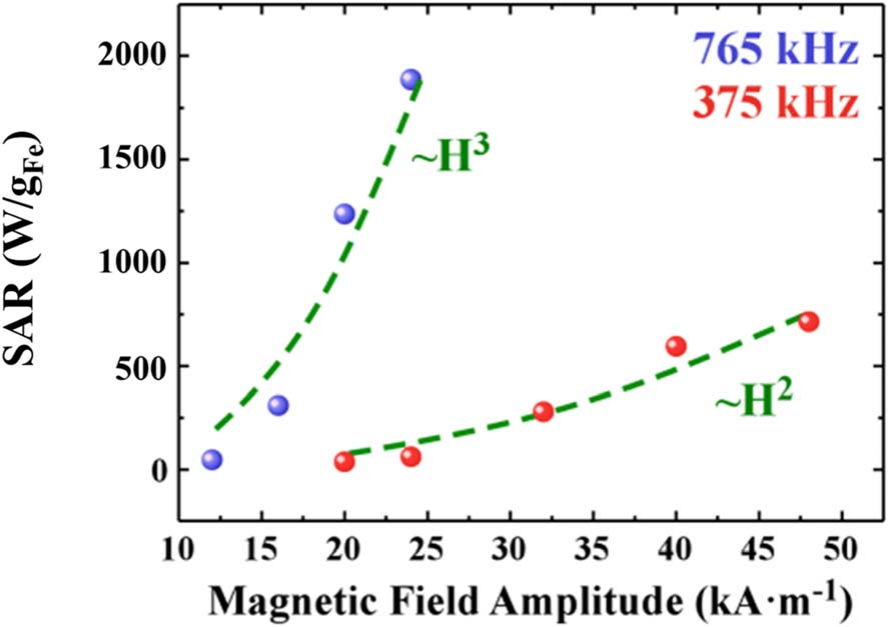
SAR index dependence on magnetic field amplitude and frequency of single domain MNPs. SAR data points are presented with red and blue color and are fitted with best fitting curve (green dotted line) for two typical frequency values used in MPH, namely 375 and 765 kHz, respectively.^40^

Then the SAR in watt per magnetic material mass would be given by 
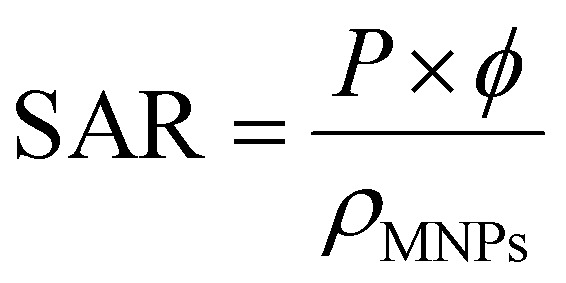
 where *ϕ* is the magnetic nanoparticles volume fraction inside the magnetic fluid and *ρ*_MNPs_ is the MNPs density. It is important to point out that, although according to eqn (38) the heating efficiency depends on the magnetic field amplitude squared, a dependence on the amplitude cube has been found in literature^74,96,97^ for higher frequencies as it is depicted in Fig. 13. In addition, the power dissipation and thus the SAR of the MNPs is maximized for *ωτ* = 1. This condition is satisfied when the relaxation time of the nanoparticles is equal to the AC field time constant and determines the critical frequency of the system.^98,99^

**Fig. 14:**
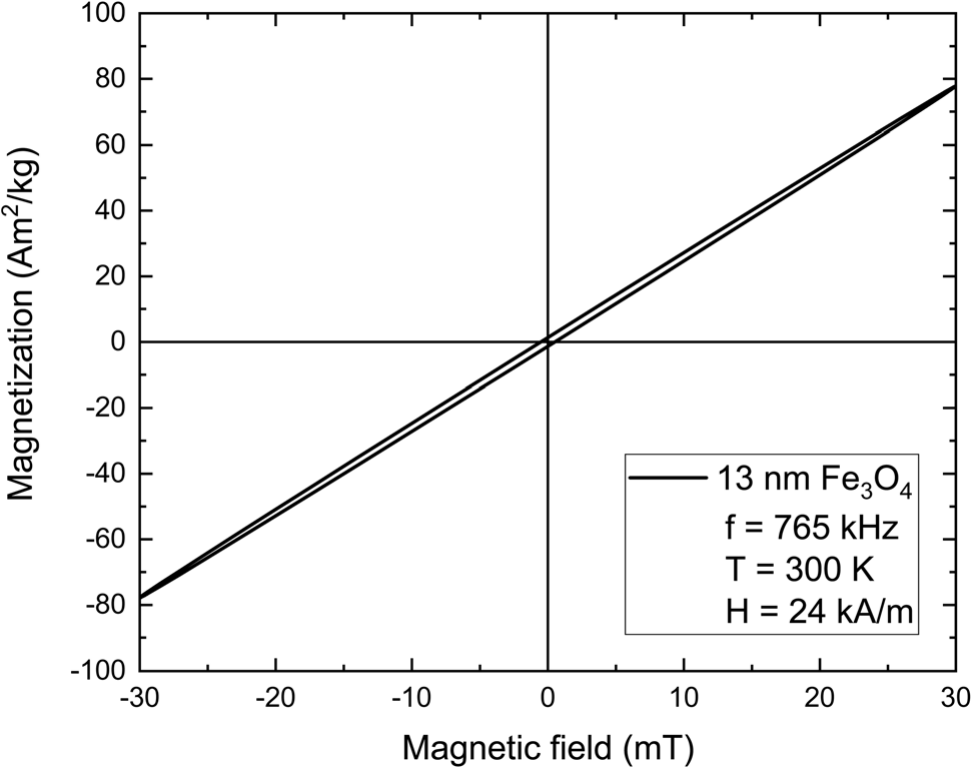
Hysteresis loop for non-interacting magnetite nanoparticles of diameter 13 nm. The applied magnetic field amplitude and the bulk saturation magnetization are equal to 24 kA m^−1^ (30 mT) and 91 A m^2^ kg^−1^ (480 kA m^−1^). From the shape of the loop, it is revealed that the linear relationship between magnetization and the applied magnetic field is reasonable approximation.

In several articles, eqn (38) is defined as applying to “relaxation losses” of superparamagnetic MNPs. In these articles, “relaxation losses” are opposed – as if it was a different process – to the “hysteresis losses” of ferromagnetic NPs. This distinction can lead to confusion as all the losses, whether the MNPs are in the superparamagnetic regime or in the ferromagnetic regime (>20 nm), are always “hysteresis losses” insofar as they are simply given by the hysteresis loop area. By drawing the parametric plot (*H*(*t*), *M*(*t*)) we can find the hysteresis loop. Basic mathematics indicates that eqn (34) and (36) correspond to the parametric equation of an ellipse in the (*H*, *M*) plane that forms an angle between its long axis and the abscise axis. A typical hysteresis loop resulted after employing LRT to a specific MNPs system is showing in Fig. 14. Thus, LRT is simply one model among several that aims to calculate the hysteresis loop area and shape when the magnetic response is linear with the applied magnetic field. In^63^ the authors suggested putting an end to the distinction between hysteresis losses and relaxation losses and, rather, making a distinction between different kinds of models aiming at calculating the hysteresis area.

In real systems a magnetic fluid contains nanoparticles of different sizes that can be reasonably described by a log-normal distribution:^72,93–96^
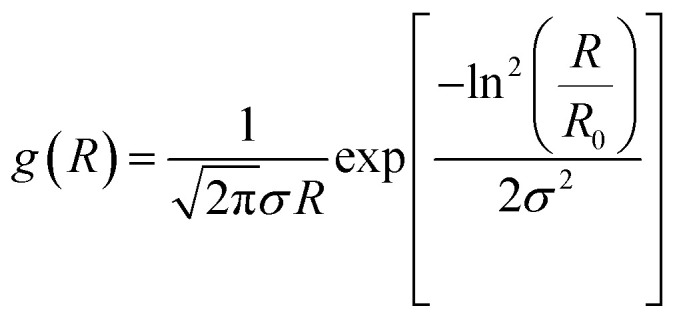
 with normalization condition 
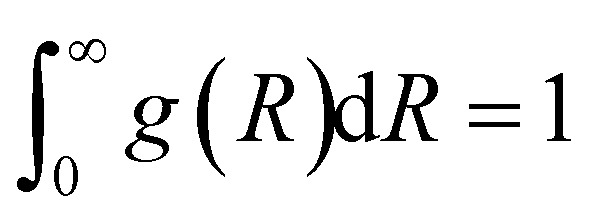
 where *σ* is the standard deviation, *R*_0_ the median of ln *R* and the mean nanoparticle radius *R*_m_ is given by: 
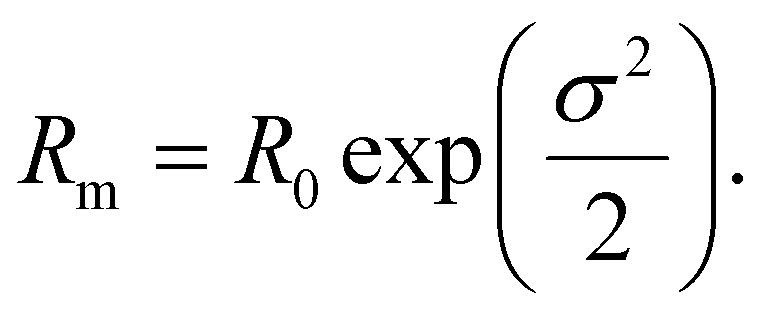
 The volumetric power dissipation *P̄* of a polydispersion is obtained by integrating over the distribution function: 
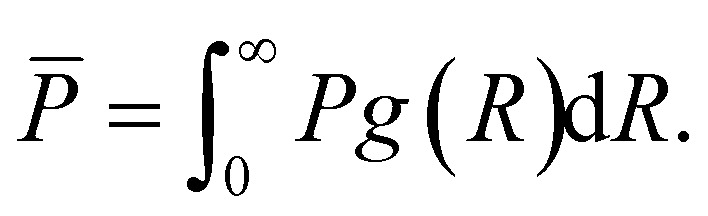
 The size distribution of nanoparticles should be considered in the calculation of power dissipation, in order to avoid overestimated results. The degradation of power dissipation for increasing standard deviation, implies that narrow size distributions are desired for optimum heating efficiency.^97–100^ It is important to point out that the effective relaxation time depends exponentially on the particle volume and anisotropy in systems where Néel relaxation is the primary heating mechanism. Thus, the influence of both parameters on heating efficiency is very significant. Tailoring highly anisotropic nanoparticles allows for the tuning of effective relaxation time on higher levels, enabling highly efficient systems at lower frequencies and smaller sizes.^101–103^ Though LRT becomes inaccurate under strong interparticle interactions and spatial magnetization complexities, it offers simplicity and speed for estimations and evaluations in SPM systems where dipolar interactions do not dominate.

## Conclusions

A thorough understanding and accurate assessment of the intrinsic magnetic properties of nanoscale systems are crucial for both fundamental research and application-driven design. In this context, the magnetic behavior of individual nanoparticles can be effectively described using simplified analytical models, such as the macrospin approximation and double well (DW) models. These approaches assume that the entire nanoparticle behaves as a single giant magnetic moment, capable of switching between two energy minima (metastable states) separated by an energy barrier. The thermally activated magnetization reversals between these states, characterized by saddle points in the energy landscape, provide a theoretical basis for estimating the magnetization of a single nanoparticle. Additionally, numerical techniques, such as Monte Carlo simulations, are employed to refine these models and extend their predictive capabilities.

However, when dealing with many-body systems, where interactions between multiple nanoparticles become significant, analytical energy minimization techniques are no longer sufficient. Instead, micromagnetic simulations and dynamical models are required to capture the complex magnetization dynamics. A widely used approach is the LLG equation, which governs the precessional motion of magnetization in interacting nanoparticle systems. By utilizing micromagnetic simulation software—such as OOMMF, MuMax, NMAG, and MicroMagus—hysteresis loops of nanoparticle assemblies can be computed, enabling the evaluation of their collective magnetic properties.

In the case of MPH applications, the presence of an externally applied AMF introduces additional complexity to the magnetization dynamics. To optimize heating efficiency for therapeutic use, the arrangement and interactions of nanoparticles under a dynamic magnetic field must be carefully examined. Molecular dynamics simulations complement micromagnetic models by providing insights into nanoparticle alignment and aggregation under field influence, which directly affects their heating performance. Moreover, in LRT, the dominant relaxation mechanism depends on factors such as nanoparticle size, magnetic anisotropy, and surrounding medium viscosity. Optimizing these relaxation processes is essential to maximize heat dissipation while ensuring efficient energy absorption under clinical operating conditions during a magnetic hyperthermia scheme.

Due to the inherent complexity of magnetization dynamics in these systems, only relatively simple cases—such as single-domain nanoparticles described by the macrospin approximation—can be solved analytically. In contrast, micromagnetic simulations offer a more realistic and comprehensive representation by incorporating all relevant energy contributions, including exchange energy, anisotropy energy, Zeeman energy, magnetostatic interactions, and dipolar interactions. This enables precise predictions of the spatial and temporal evolution of magnetization under external stimuli.

Micromagnetic modeling continues to be an invaluable tool for advancing nanoscale magnetic research, bridging the gap between theoretical predictions and experimental observations. The future of numerical simulations in this field lies in the development of multiphysics approaches, integrating magnetization dynamics with additional physical processes such as heat transfer, fluid dynamics, and mechanical stress effects. While current software solutions are making strides toward such integration, a fully optimized, general-purpose multiphysics package remains an ongoing challenge. Enhancing user-friendly interfaces will also be essential for facilitating the use of micromagnetic simulations in both fundamental research and applied technologies.

Looking ahead, numerical simulations are expected to further expand their role as the primary tool for designing and optimizing magnetic nanoparticles, particularly for biomedical applications such as MPH. The increasing interest in nanoscale and many-body magnetic systems underscores the necessity of simulation-driven design, enabling the tailoring of magnetic properties for next-generation nanoparticle-based therapies. Beyond the current state-of-the-art, future efforts are anticipated to focus on the development of comprehensive multiphysics frameworks capable of simultaneously modeling magnetization dynamics, heat transfer, fluid flow, and even biological interactions at cellular and tissue levels. This holistic approach will allow for the accurate prediction of nanoparticle behavior under realistic physiological conditions, bridging the gap between theoretical models and clinical applications. Furthermore, coupling micromagnetic simulations with experimental feedback loops and machine learning techniques is expected to significantly accelerate the discovery and optimization of MNPs, delivering customized solutions tailored to specific patient needs and tumor microenvironments. Ultimately, such advances will not only enhance the therapeutic efficiency and safety of MPH treatments but will also open new pathways in the broader field of nanomedicine, establishing numerical simulations as a cornerstone in the rational design of multifunctional nanomaterials for biomedical innovation.

## Conflicts of interest

There are no conflicts to declare.

## Data Availability

Data for this article, including origin data are available at the site of the MagnaCharta Lab of the Physics Department of Aristotle University of Thessaloniki at https://magnacharta.auth.gr/.
