# Correction: A comprehensive analysis of nanomagnetism models for the evaluation of particle energy in magnetic hyperthermia

**DOI:** 10.1039/d6na90021f

**Published:** 2026-03-16

**Authors:** N. Maniotis, M. Maragakis, N. Vordos

**Affiliations:** a Department of Physics, Aristotle University of Thessaloniki Thessaloniki Greece nimaniot@physics.auth.gr; b Department of Physics, Democritus University of Thrace Kavala Greece

## Abstract

Correction for ‘A comprehensive analysis of nanomagnetism models for the evaluation of particle energy in magnetic hyperthermia’ by N. Maniotis *et al.*, *Nanoscale Adv.*, 2025, **7**, 4252–4269, https://doi.org/10.1039/D5NA00258C.

The authors regret that the *ξ* value in the Fig. 2 caption was stated incorrectly in the manuscript. The correct value is included here along with the figure and the caption.

**Fig. 2:**
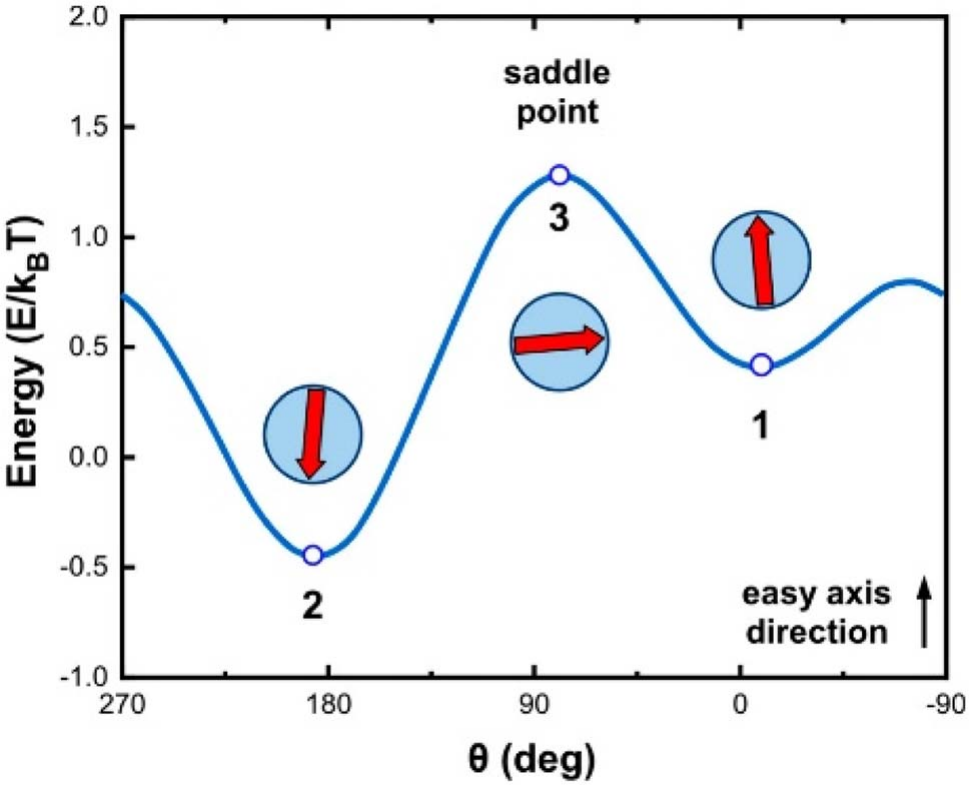
Schematic representation of the energy landscape variation with the “easy” axis-magnetic moment angle (double-well approximation) for *ξ* = −0.5 and *ϕ* = 30°. The red arrows show the magnetic moment orientation. When *ξ* ≠ 0, *θ*_1_, *θ*_2_, and *θ*_3_ values deviate slightly from 0, 180 and 90° which correspond to parallel antiparallel and perpendicular orientation, with respect to the “easy” axis, respectively. Although this deviation exists, we can approximately employ 0 and 180° as the minimum energy positions without a significant deviation from the realistic experimental behavior.^45^

The Royal Society of Chemistry apologises for these errors and any consequent inconvenience to authors and readers.

